# *In vitro* activity of zosurabalpin against a global set of carbapenem-resistant-*A. baumannii* clinical isolates

**DOI:** 10.1128/aac.00727-26

**Published:** 2026-09-16

**Authors:** Stefano Mancini, Helena M. B. Seth-Smith, Klara Haldimann, Jacob Moran-Gilad, Elif Aktas, Öznur Güneş, Mohammedaman Mama Hussen, Mark Willcox, Shakeel Shahzad, Muhammad Ali Syed, Natalia Kolesnik-Goldmann, Tim Roloff, Oliver Nolte, Adrian Egli

**Affiliations:** 1 Institute of Medical Microbiology, University Zurich 27217 , Zurich, Switzerland; 2 SIB Swiss Institute of Bioinformatics, Zurich, Switzerland; 3 Clinical Microbiology Laboratory, Hadassah Hebrew University Medical Center, Jerusalem, Israel; 4 SBÜ Hamidiye Etfal Training and Research Hospital, Istanbul, Türkiye; 5 Goba Referral Hospital, Madda Walabu University 233750, Robe, Ethiopia; 6 School of Optometry and Vision Science, University of New South Wales 7800, Sydney, Australia; 7 Department of Microbiology, University of Haripur 384987, Haripur, Pakistan; Universita degli Studi di Roma La Sapienza, Rome, Italy

**Keywords:** novel antibiotic class, carbapenem-resistant-*Acinetobacter baumannii*, zosurabalpin

## Abstract

The limited treatment options for carbapenem-resistant *Acinetobacter baumannii* (CRAB)-invasive infections highlight the urgent need for novel therapeutic agents. Zosurabalpin is a first-in-class tethered macrocyclic-peptide targeting the LptB₂FGC-complex. In this study, we evaluated the *in vitro* activity of zosurabalpin a1gainst a diverse global set of CRAB clinical isolates. We compared zosurabalpin’s activity with currently available antibiotics, including colistin, cefiderocol, sulbactam-durlobactam, as well as rifabutin. Additionally, we assessed different AST methods to support reliable zosurabalpin susceptibility testing. We included a total of 304 CRAB clinical isolates from Switzerland, Israel, Turkey, Ethiopia, Pakistan, and Australia. Among them, 297 carried plasmid-borne-carbapenemases (236 OXA-type-producers, 60 NDM-type-producers alone or in combination with OXA-type-carbapenemases, and 1 GES-14-producer), 3 were GES-ESBL-producers, and 4 isolates were without acquired ESBL/carbapenemases. We determined zosurabalpin minimal inhibitory concentrations (MICs) by broth microdilution using cation-adjusted Mueller-Hinton broth (CA-MHB) supplemented with 20% heat-inactivated horse serum, in accordance with CLSI recommendations, as well as using plain CA-MHB, with reading at substantial reduction (80% growth inhibition). We determined MICs of the remaining antibiotics according to EUCAST methods: colistin and sulbactam-durlobactam (with fixed durlobactam concentration of 4 mg/L) with CA-MHB, cefiderocol with iron-depleted CA-MHB, and rifabutin with RPMI. The zosurabalpin MIC distribution using the CLSI-based method ranged from ≤0.032 to 2 mg/L (MIC_90_ = 0.5 mg/L), with no CRAB isolates showing MICs displaying MICs suggestive of high-level acquired resistance. A single isolate with MIC = 2 mg/L harbored a 13-amino-acid deletion in *lptD* (position 692), potentially affecting outer-membrane LPS biogenesis. MIC_90_ values for cefiderocol, sulbactam–durlobactam, colistin, and rifabutin were 8, 16, 1, and 2 mg/L, respectively. Zosurabalpin showed no cross-resistance with any of the tested antimicrobials. Zosurabalpin MICs in CRAB isolates were generally low, including metallo-β-lactamase-producing-CRAB. The low MICs show a high potential to overcome current treatment limitations; however, mutations in *lptD* may contribute to resistance emergence and should be further studied.

## INTRODUCTION

Over the past decades, *Acinetobacter baumannii* has emerged as a major nosocomial pathogen worldwide. Its remarkable genomic plasticity facilitates acquisition of antimicrobial resistance (AMR) determinants, particularly in high-risk hospital environments (1). Consequently, carbapenem-resistant *A. baumannii* (CRAB) has become associated with severe healthcare-associated infections, including pneumonia, bloodstream infections, wound infections, and device-related infections, resulting in substantial morbidity and mortality (2). Carbapenem resistance is predominantly mediated by acquired class D oxacillinases (e.g., OXA-23, OXA-24/40, OXA-58), whereas class A and B carbapenemases occur less frequently (2). Therapeutic options for CRAB remain limited. Agents such as polymyxins, tigecycline/eravacycline, cefiderocol, and sulbactam-durlobactam (sul-dur) show variable efficacy and may be compromised by toxicity or emerging resistance (3–6). NDM-producing CRAB isolates are particularly problematic, as durlobactam lacks activity against metallo-β-lactamases and cefiderocol susceptibility is generally reduced (7). Rifabutin, an “old” antibiotic traditionally considered ineffective against Gram-negative bacteria, has recently re-emerged as a potential therapeutic option for the treatment of CRAB infections, and an intravenous formulation (BV100) is currently being evaluated in a Phase III clinical trial (https://clinicaltrials.gov/study/NCT07326540) (8).

Zosurabalpin (RG6006), a novel tethered macrocyclic peptide developed by Roche, represents the first member of a new antibiotic class targeting the lipopolysaccharide transport (Lpt) system. By inhibiting the inner membrane LptB₂FGC transporter, zosurabalpin blocks lipopolysaccharide export and induces bacterial death (9, 10). Phase I studies showed favorable safety and pharmacokinetic profiles, and the compound is currently progressing toward advanced clinical evaluation (10). Susceptibility testing of zosurabalpin remains challenging because standard broth microdilution (BMD) in cation-adjusted Mueller–Hinton broth (CA-MHB) frequently produces pronounced trailing endpoints. The addition of 20% heat-inactivated horse serum (hoS) substantially improves endpoint readability and is currently recommended by CLSI.

In this study, we evaluated the activity of zosurabalpin against a geographically and genetically diverse collection of 304 CRAB isolates. Zosurabalpin susceptibility distributions were compared with those of cefiderocol, sulbactam-durlobactam, colistin, and rifabutin. In addition, we evaluated different susceptibility testing approaches, including CLSI reference BMD, CA-MHB substantial reduction (SR) endpoint reading, and disc diffusion (DD).

## MATERIALS AND METHODS

### Strain collection

A total of 304 non-duplicate CRAB isolates from Switzerland, Pakistan, Israel, Turkey, Ethiopia, and Australia were included (Table S1). The collection comprised 297 carbapenemase-producing isolates, mainly harboring blaOXA-23, blaOXA-72, or NDM variants, 3 GES-11 ESBL producers, and 4 carbapenem-resistant isolates lacking acquired carbapenemase or ESBL genes

### MIC determination

Zosurabalpin was kindly provided by F. Hoffmann-La Roche Ltd (Basel, Switzerland). Zosurabalpin MICs were determined in duplicate by BMD using two approaches: (i) according to CLSI guidelines, using CA-MHB supplemented with heat-inactivated 20% hoS (Horse Serum Donor Herd, USA origin, Heat inactivated, sterile-filtered; Sigma-Aldrich, reference H1138) (hereafter CLSI standard method) (11); (ii) using CA-MHB without hoS and reading at substantial reduction (SR, corresponding in our MIC readings with approximately 80% [±10%] growth inhibition, ignoring trailing effect and regrowth at higher concentrations; hereafter, CA-MHB, SR method). In cases where MICs differed between duplicates, which occurred in approximately 30% of instances (with ~70% identical results, ~25% within ±1 dilution, and 5% within ±2 dilutions), the MIC of the first duplicate was consistently recorded for the final analysis. MICs of the remaining antibiotics were determined according to the EUCAST method: colistin (Sigma Aldrich, Israel) and sulbactam-durlobactam (sulbactam from MCE MedChemExpress, USA; durlobactam from AdooQ Bioscience, USA, used at a fixed concentration of 4 mg/L) with CA-MHB, cefiderocol with iron-depleted CA-MHB (Bruker, Germany) (12), and rifabutin (Sigma Aldrich, USA) with RPMI 1640 (BioConcept, Switzerland), +10% FCS (Sigma Aldrich, USA) (8). *Pseudomonas aeruginosa* ATCC27853 and *Acinetobacter baumannii* NCTC13304 were used as internal quality controls (Fig. S1). The antimicrobial agents (except for colistin) were prepared as a stock solution of 20 mM in DMSO (although CLSI guidelines suggest dilution in water). Colistin was diluted in sterile water. Aliquots were stored at −80°C. Twofold serial dilutions of the antimicrobials were prepared in the appropriate media in a 2 mL deep-well plate by using the Viaflo 96 pipette robot (Integra, Switzerland). From the deep well plate, 97 µL was dispensed (Viaflo 96) into each 96-well microtiter plate. Freshly grown colonies on COS agar (bioMérieux SA, France) incubated at 35°C ± 2°C for 18–24 h were used to adjust turbidity to 0.5 McF standard. The suspension was further diluted in sterile water to reach the final density in the well of approximately 5 × 10⁵ CFU/mL. Subsequently, 3 µL of the standardized bacterial inoculum was added (Viaflo 96) to each well, resulting in a final volume of 100 µL per well. On each test day, the quality control isolates *P. aeruginosa* ATCC27853 and *A. baumannii* NCTC13304 were included. Inoculum CFU control of approximately 25% of isolates was carried out to verify the correct density. Following incubation at 35°C ± 2°C for 20–24 h, wells were examined visually for turbidity. The MIC was determined according to CLSI (zosurabalpin) or EUCAST guidelines (13).

#### Disc diffusion

The Kirby–Bauer disc diffusion (DD) method was performed on MH agar plates following EUCAST guidelines, using 5-µg zosurabalpin discs (Liofilchem, Italy) (14).

### Whole-genome sequencing

Bacterial genomic DNA was extracted using either the DNeasy UltraClean Microbial Kit (Qiagen, Hilden, Germany) or the Maxwell RSC Genomic DNA Kit (Promega, Madison, WI, USA), following the manufacturers’ instructions. Library preparation was performed with the QIAGEN QIASeq FX kit (Qiagen, Hilden, Germany). Paired-end sequencing (2 × 150 bp) of DNA libraries was conducted using an Illumina MiSeq or NextSeq1000 platform (Illumina, San Diego, CA, USA). Bioinformatic analysis for quality control and assembly was performed using the IMMense pipeline (15). All genome sequences generated at the Institute of Medical Microbiology, University of Zurich, were deposited in the European Nucleotide Archive; some had been previously submitted under project accession PRJEB62871 (Ab01–Ab100) (16), while the remaining sequences were deposited under accession PRJEB108761.

### Bioinformatics analysis and detection of β-lactam resistance genes

Genome assemblies were typed in Ridom Seqsphere + v10.5.4 by multi-locus sequence typing (MLST) according to the Pasteur (sequence type, ST) scheme and by core genome multi-locus sequence typing (cgMLST) (17). For resistance gene analysis, assemblies were analyzed using NCBI AMRFinderPlus v1.3.1 (18) within Ridom Seqsphere+. Additional potential genomic determinants associated with colistin resistance were investigated from the assemblies by alignment in Aliview v1.27 (http://dx.doi.org/10.1093/bioinformatics/btu531) and comparative analysis, with mapping against reference data using bwa (10.1093/bioinformatics/btp324) and visualization in Artemis (10.1093/bioinformatics/16.10.944) where necessary. This included *pmrA/pmrB*, *eptA* (the *pmrC* homolog), and *lpxA*, *lpxC*, and *lpxD* (19) in isolates exhibiting colistin-resistant MICs (>4 mg/L), all belonging to ST2. Isolate CRABn301, also classified as ST2, displayed a colistin MIC of 0.25 mg/L. In addition, the genomic region upstream of *eptA* was examined to confirm or exclude the presence of the insertion sequence IS*Aba*1. Genomic mutations potentially associated with resistance to sulbactam–durlobactam (MICs > 8 mg/L) were investigated in all CRAB isolates lacking NDM-type carbapenemases (*n* = 7). Specifically, the *pbp3* (*ftsI*), *pbpB1*, and *pbpA* genes were analyzed and compared with corresponding sequences from isolates of the same sequence type (ST) exhibiting low sulbactam–durlobactam MICs (20). The following isolate pairs were compared: CRABn265 (ST592, OXA-72) vs CRABn33 (ST78, OXA-72); CRABn8 and CRABn199 vs CRABn197 (all ST2); CRABn195 and CRABn305 vs CRABn193 (ST85); and CRABn291 and CRABn292 vs CRABn46 (ST570). In addition, targeted analyses of *lptF*, *lptG*, *lptM*, and *lptD* were performed in four CRAB isolates exhibiting elevated zosurabalpin MICs of 1 mg/L (*n* = 3; CRABn20, CRABn86, and CRABn226) or 2 mg/L (*n* = 1; CRABn2), as well as in 10 additional isolates with zosurabalpin MICs at or below the tentative ECOFF (CRABn26, CRABn34, CRABn37, CRABn51, CRABn106, CRABn152, CRABn184, CRABn256, CRABn282, CRABn295, and CRABn298), representing diverse sequence types and β-lactam resistance mechanisms. All sequences were compared with that of CRABn183 (ST2, MIC 0.5 mg/L). Previous studies have shown that mutations in these genes, as well as the presence of insertion sequences such as IS*Aba*1 upstream of the efflux pump regulator genes *adeS/adeR*, may be associated with reduced susceptibility to zosurabalpin (10). Accordingly, the genomic region upstream of *adeS/adeR*, as well as mutations within *adeS*, was examined.

Finally, *A. baumannii* genomes were downloaded from NCBI Pathogen Detection Isolate browser on 27 April 2026 and filtered for the isolation source indicating blood, urine, respiratory or environmental origin, based on the list in Appendix A, resulting in 9,142 genomes. The respective genome assemblies were screened for genes *lptF, lptG,* and *lpxM* by aligning reference loci, including flanking regions of 200 bp, of the reference strain *A. baumannii* ATCC 17978, to each assembly using minimap2. Nucleotide substitutions and indels were reported relative to the start of the respective coding sequence and were subsequently applied to the reference coding sequence to reconstruct and translate the corresponding protein sequence. As a result, between 8,850 and 8,883 public *A. baumannii* genomes were analyzed (specifically 8,877 for *lptF*, 8,883 for *lptG*, and 8,850 for *lptM*). The resulting amino acid sequences of LptF, LptG, and LpxM were compared against the reference strain *A. baumannii* ATCC 17978. Amino-acid variants were classified as synonymous, missense, frameshift, or in-frame deletion variants. All variants previously associated with elevated zosurabalpin MICs by Zampaloni et al. were annotated (10). Furthermore, we flagged novel variants affecting the same amino acid positions as experimentally validated resistance-associated substitutions, as these may exert similar effects on zosurabalpin susceptibility. We also highlighted variants causing amino acid alterations within a maximum distance of three amino acids from any previously validated Zampaloni et al. resistance site. Frameshift mutations predicted to disrupt gene function were also identified as potential resistance candidates. The prevalence of all detected variants, including absolute numbers and percentages across all analyzed genomes, is provided in Table S2.

## RESULTS

### Genetic diversity of the CRAB collection

**Fig 1:**
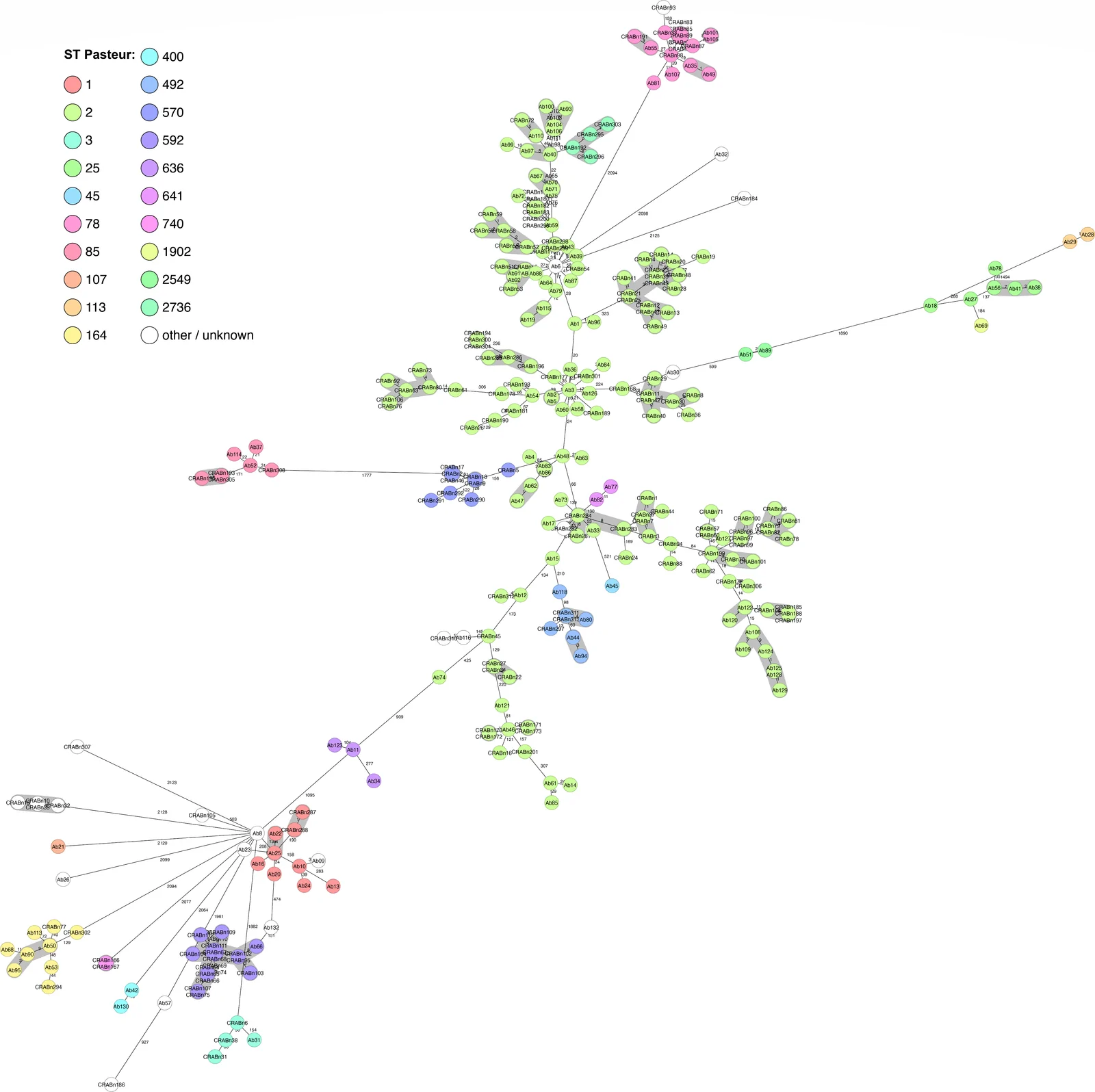
Minimum Spanning Tree showing the core genome MLST phylogenetic relationship between all isolates. 304 isolates are shown, colored by MLST Pasteur sequence type as per the key. Allele distances between the isolates are given on the branches. Clusters as defined by a cut-off of 9 allele differences are indicated with a gray shadow background.

The CRAB collection comprised 304 isolates belonging to 43 distinct Pasteur sequence types (STs), exhibiting substantial population diversity (Fig. 1). ST2 predominated (197/304; 64.8%), followed by ST78, ST592, ST492, ST85, and ST164. Carbapenemase distribution showed strong lineage association: ST2 was mainly linked to blaOXA-23, whereas blaOXA-72 predominated in ST78 and ST636. Carbapenemase distribution showed strong lineage association: ST2 was overwhelmingly linked to *bla*_OXA-23_ (≈90%), frequently co-occurring with *armA*, whereas ST78 and ST636 were almost exclusively associated with *bla*_OXA-72_, and NDM-producing isolates clustered within a limited number of STs, particularly ST592, ST492, ST570, and ST85.

### Antimicrobial susceptibility testing and correlation with resistance mechanisms

**Fig 2:**
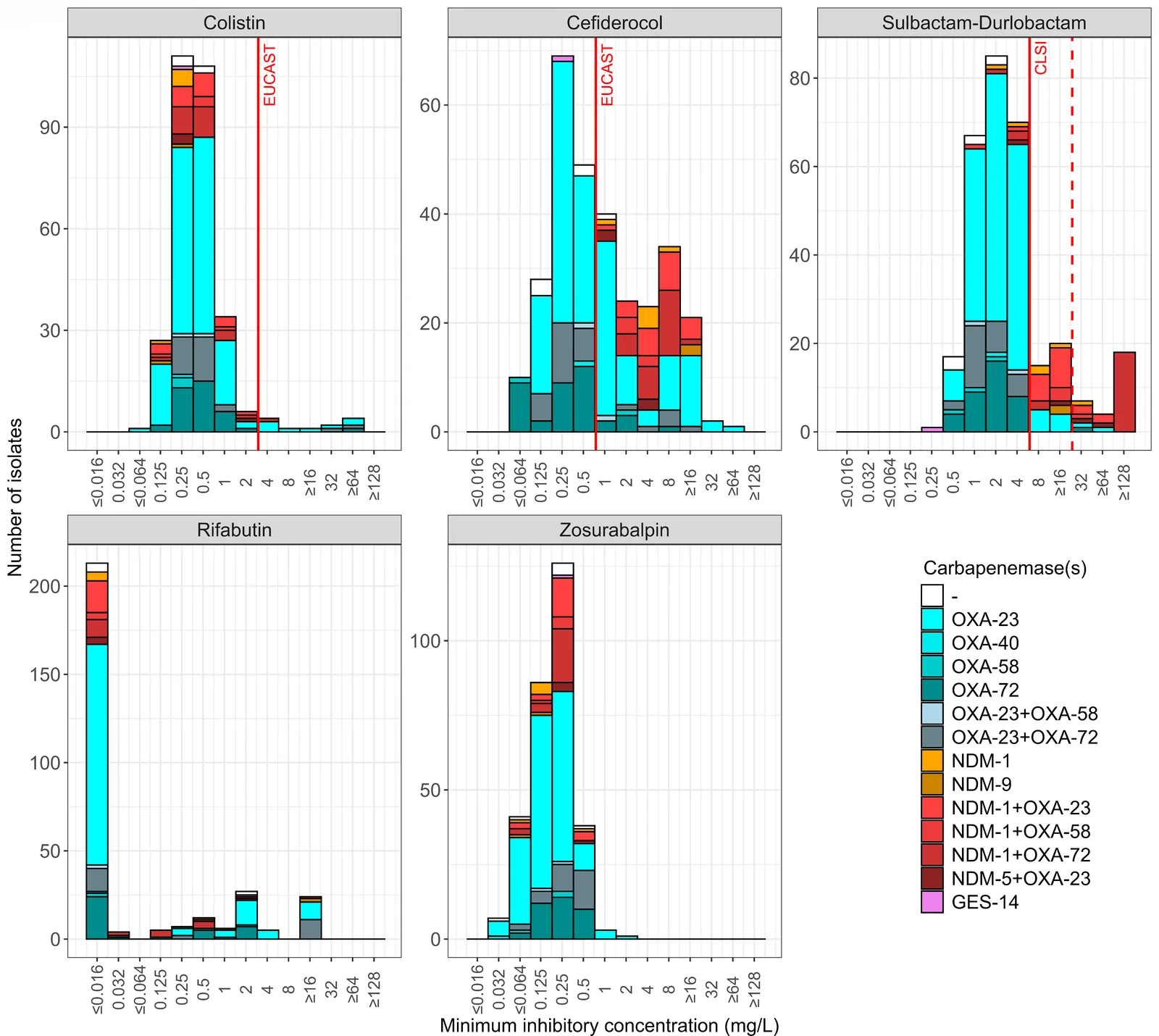
Distribution of minimum inhibitory concentrations of CRAB isolates grouped by carbapenemase type(s). Vertical solid and dashed red lines indicate the EUCAST and CLSI clinical breakpoints, respectively. Histogram bars are colored according to carbapenemases detected by WGS.

Zosurabalpin MICs determined by the CLSI reference BMD (CA-MHB supplemented with heat-inactivated 20% hoS) ranged from 0.032 to 2 mg/L. The modal value was 0.25 µg/mL, the MIC_50_ and MIC_90_ values were 0.25 and 0.5 µg/mL, respectively. Visual inspection of the MIC distribution revealed a narrow, unimodal wild-type population, supporting a tentative epidemiological cutoff value (ECOFF) of 0.5 mg/L (Fig. 2). Only 4/304 isolates (1.3%), all belonging to ST2, exhibited MICs marginally above this tentative ECOFF (three at 1 mg/L and one at 2 mg/L), with no evidence of a distinct non-wild-type subpopulation. Consistently, no mutations in *lptF*, *lptG*, or *lpxM*—previously associated with elevated MICs—were detected in any of the CRAB isolates. A stop mutation after amino acid 126 of *adeS* was identified in one of three isolates with an MIC of 1 mg/L and in the single isolate with an MIC of 2 mg/L (Table S1). Notably, a 13-amino acid deletion at position 692 of LptD was detected in an ST2 CRAB isolate exhibiting an MIC of 2 mg/L. LptD is the outer-membrane translocon for lipopolysaccharide (LPS), forming a β-barrel protein in the outer membrane. Together with LptE, it constitutes the LptDE complex, which mediates insertion of LPS into the outer leaflet of the outer membrane (21). To further investigate the contribution of these genes to zosurabalpin susceptibility, *lptF, lptG, lpxM*, and *lptD* were analyzed in an additional panel of 10 CRAB isolates with wild-type zosurabalpin MICs representing diverse sequence types and β-lactam resistance mechanisms. The panel included ST107 *bla*_OXA-40_, ST114 *bla*_GES-14_, ST15 *bla*_OXA-58_, three ST2 *bla*_OXA-23_ isolates with different colistin susceptibility profiles, ST2 *bla*_NDM-5_ *bla*_OXA-23_, ST78 *bla*_OXA-72_, one isolate of unknown ST carrying *bla*_NDM-1_, ST570 *bla*_NDM-1_ *bla*_OXA-23_, and ST740 *bla*_NDM-9_. This panel was selected to maximize genetic diversity and encompass a broad range of resistance mechanisms. Several amino acid substitutions were identified in *lptF*, *lptG*, and *lpxM*; however, none corresponded to the resistance-associated mutations previously described by Zampaloni et al. (10) Likewise, *lptD* exhibited considerable sequence variability, including several small insertions and deletions, but no frameshift mutations were detected. Taken together, these findings indicate that naturally occurring sequence variation in *lptF*, *lptG*, *lpxM*, and *lptD* is common among CRAB isolates but, by itself, does not appear to confer reduced susceptibility to zosurabalpin. While specific mutations in these genes have previously been linked to elevated zosurabalpin MICs, the functional relevance of the sequence diversity observed in *lptD* remains to be elucidated.

**Fig 3:**
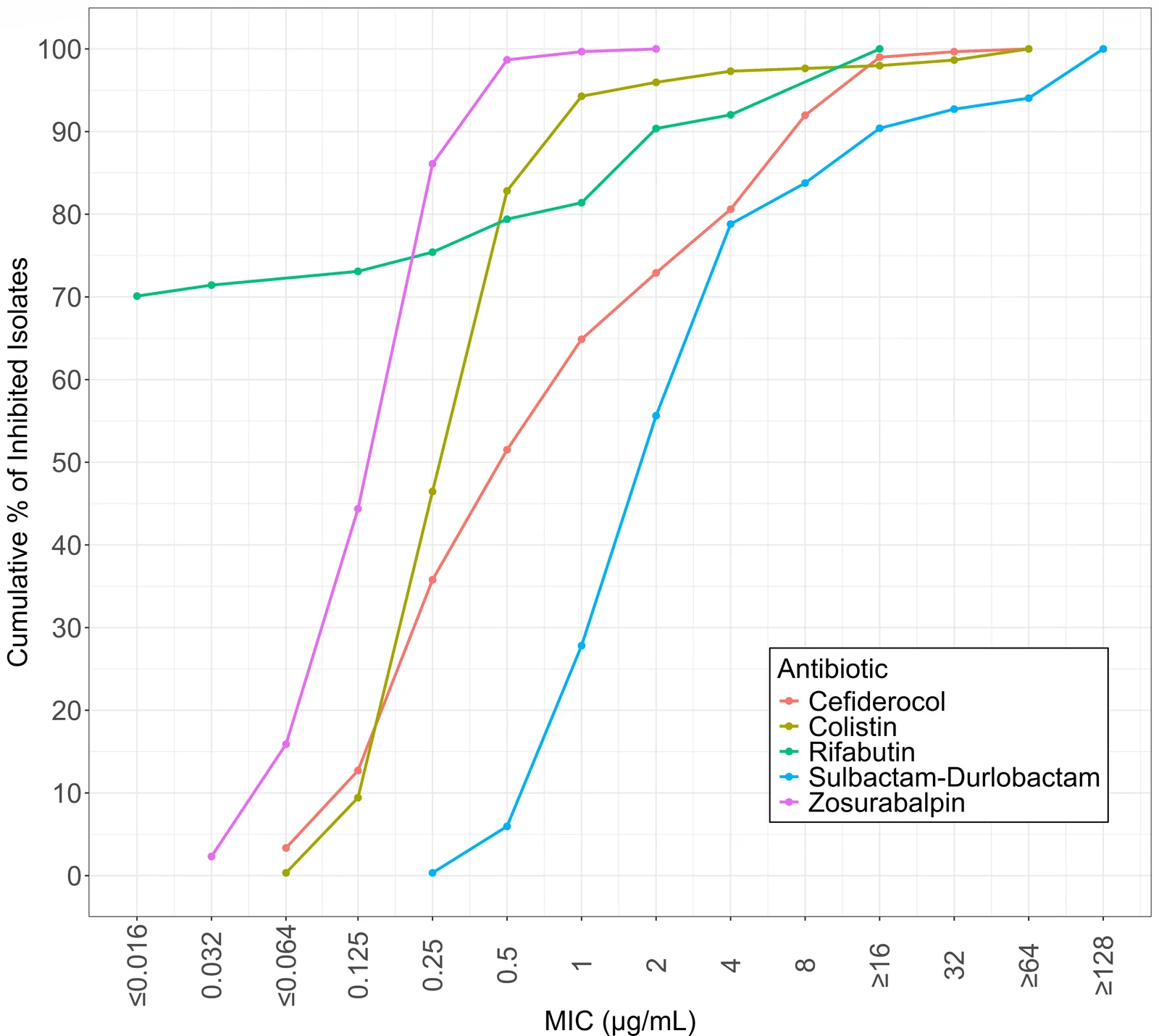
*In vitro* activity of zosurabalpin against 304 CRAB isolates. *In vitro* MIC activity of zosurabalpin against 304 carbapenem-resistant *A. baumannii* clinical isolates shown as the cumulative percentage (MIC_90_: zosurabalpin = 0.5 µg/mL; cefiderocol = 8 µg/mL; colistin = 1 µg/mL; rifabutin = 2 µg/mL; sulbactam-durlobactam = 16 µg/mL).

**Fig 4:**
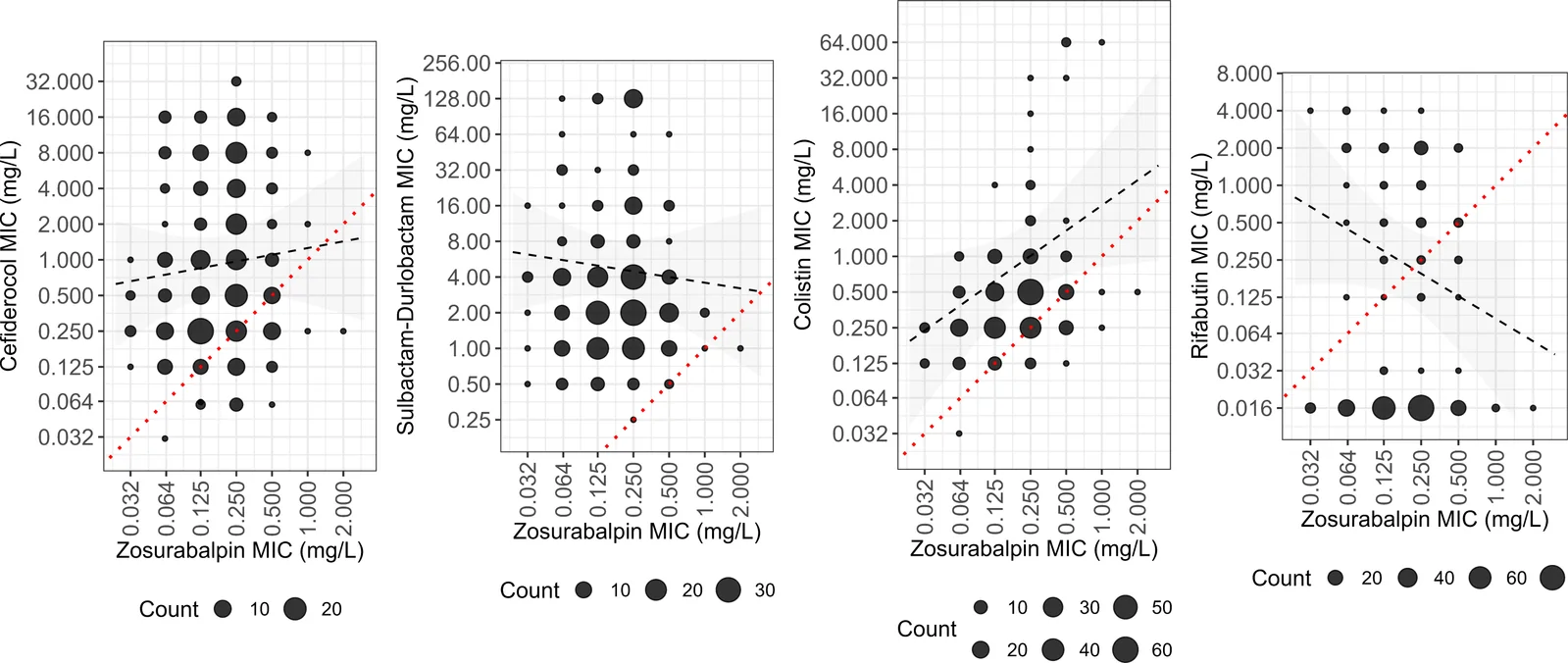
Correlation between zosurabalpin MICs determined with the CLSI standard method and MICs of cefiderocol, sulbactam-durlobactam, colistin, and rifabutin. The dotted red lines represent the lines of identity, while the black dashed lines show the regression lines.

MIC_90_ values for cefiderocol, sulbactam–durlobactam, colistin, and rifabutin were 8, 16, 1, and 2 µg/mL, respectively (Fig. 3). Importantly, higher zosurabalpin MICs were not associated with systematically higher MICs to any comparator antibiotic, suggesting that reduced susceptibility to zosurabalpin occurs independently of resistance to currently available agents (Fig. 4).

Colistin MICs were predominantly within the susceptible range (Table S1), with only 12 isolates exhibiting MICs above the clinical breakpoint of 2 mg/L. No plasmid-mediated *mcr* genes were detected in any isolate, and the majority of colistin-resistant isolates (8/12) carried mutations in the *pmrABC* locus (22). Notably, the PmrB T138A substitution was identified in four isolates. Moreover, a point mutation in *eptA*/*pmrC* leading to the amino acid substitution R109H was detected in two isolates. In 1 isolate, an IS*Aba1* insertion at codon 71 caused truncation of the N-terminus of EptA. This finding is unexpected, as elevated colistin MICs in *A. baumannii* have generally been associated with increased *eptA* expression rather than loss of EptA function (6). However, the same isolate also harbored two mutations in *pmrB*, which may have resulted in constitutive activation of the PmrAB two-component system and subsequent overexpression of PmrC (EptA homolog), thereby providing an alternative explanation for the elevated colistin MIC.

Cefiderocol susceptibility was highly heterogeneous, with MICs ranging from ≤0.064 to ≥64 mg/L. As expected, NDM-producing isolates were associated with elevated MICs. Among the 60 NDM-positive CRAB isolates, 27 (45.0%) showed cefiderocol MICs ≥ 8 mg/L and 7 (11.7%) were resistant according to CLSI criteria (MIC ≥ 16 mg/L). However, a comparable number of isolates with MICs ≥ 8 mg/L were OXA-type producers (31 isolates), consistent with the multifactorial nature of cefiderocol resistance in *A. baumannii*. Similarly, NDM-producing CRAB isolates were associated with reduced suscpetibility or resistance to sulbactam–durlobactam: 52/60 (86.7%) had MICs ≥ 8 mg/L and 42/60 (70.0%) were resistant by CLSI criteria (MIC ≥ 16 mg/L), in line with the lack of activity of durlobactam against metallo-β-lactamases. Among CRAB isolates exhibiting MICs above the CLSI clinical breakpoint (*R* > 16 mg/L), only 12 isolates did not produce an NDM carbapenemase. While the frequently described A515V substitution in the amino acid sequence of the PBP3 target, which by itself does not confer resistance (7), was detected in many CRAB isolates (*n* = 102) displaying a wide range of sul-dur MICs (0.5–64 mg/L), the T526S mutation was identified only in four CRAB isolates producing OXA-23 (*n* = 3) or OXA-72 (*n* = 1), with MICs ranging from 8 to 32 mg/L. Importantly, while NDM-producing CRAB isolates were associated with resistance to sulbactam–durlobactam and reduced susceptibility or resistance to cefiderocol, they exhibited, wild-type-like MIC distribution for zosurabalpin.

Finally, rifabutin showed high activity against the majority of isolates, with MICs ≤ 0.016 mg/L observed for 217/304 (71.4%) CRAB isolates. Non-wild-type isolates displayed a bimodal MIC distribution, likely reflecting the accumulation of one or more resistance mechanisms, including alterations in *fhuE* or within the rifampicin resistance-determining regions (RRDRs) of *rpoB*, as well as the presence of plasmid-mediated resistance determinants such as *arr-2* and and *fhuE*^LAC-4.^ (8). Rifabutin MICs did not show a monotonic or directional association with susceptibility to any of the other antimicrobials tested.

### Correlation between different zosurabalpin MIC-BMD methods and DD

**Fig 5:**
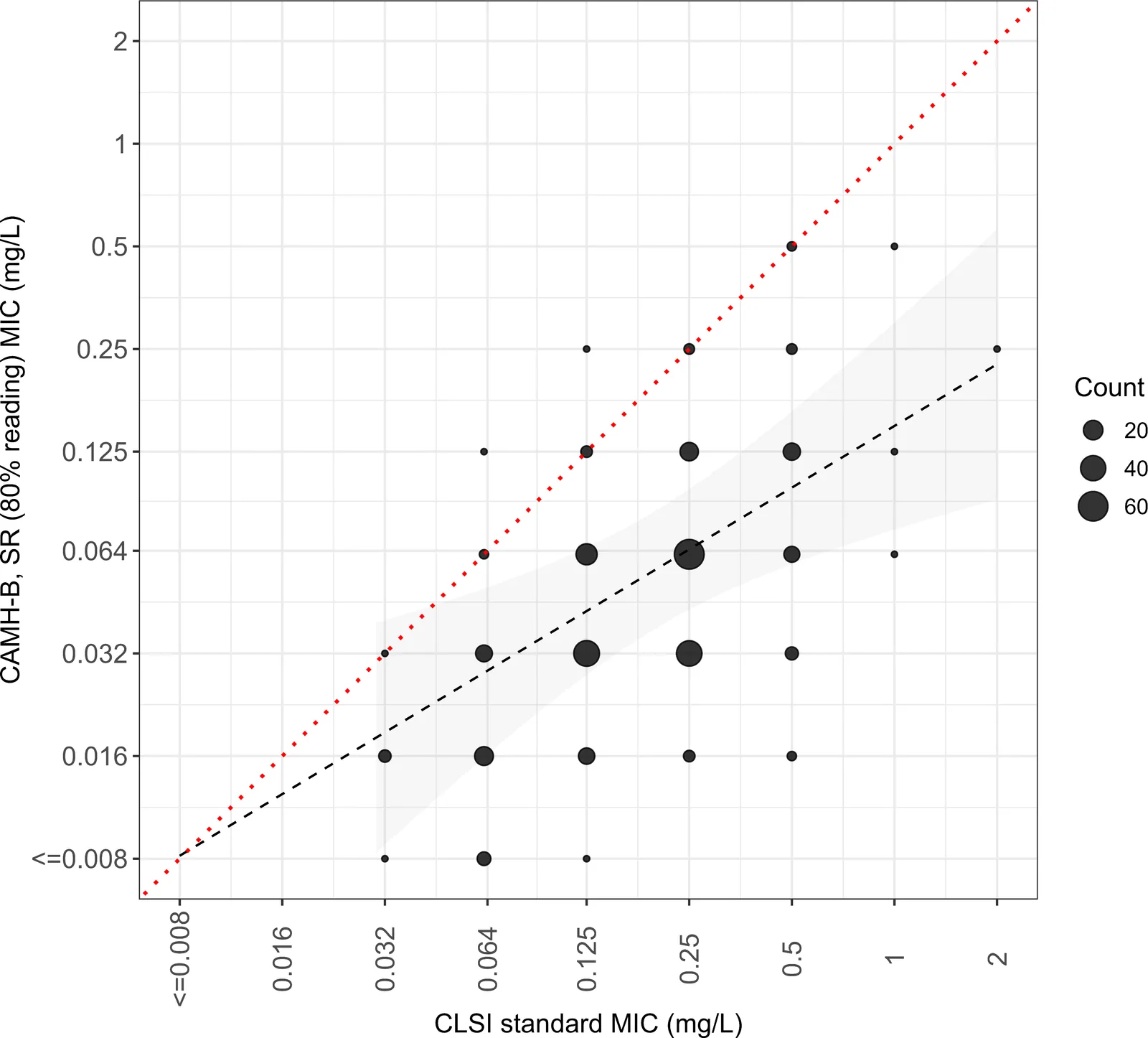
Correlation between MICs determined with the CLSI standard and CA-MHB, SR methods. The dotted red line represents the line of identity, while the black dashed line shows the regression line.

**Fig 6:**
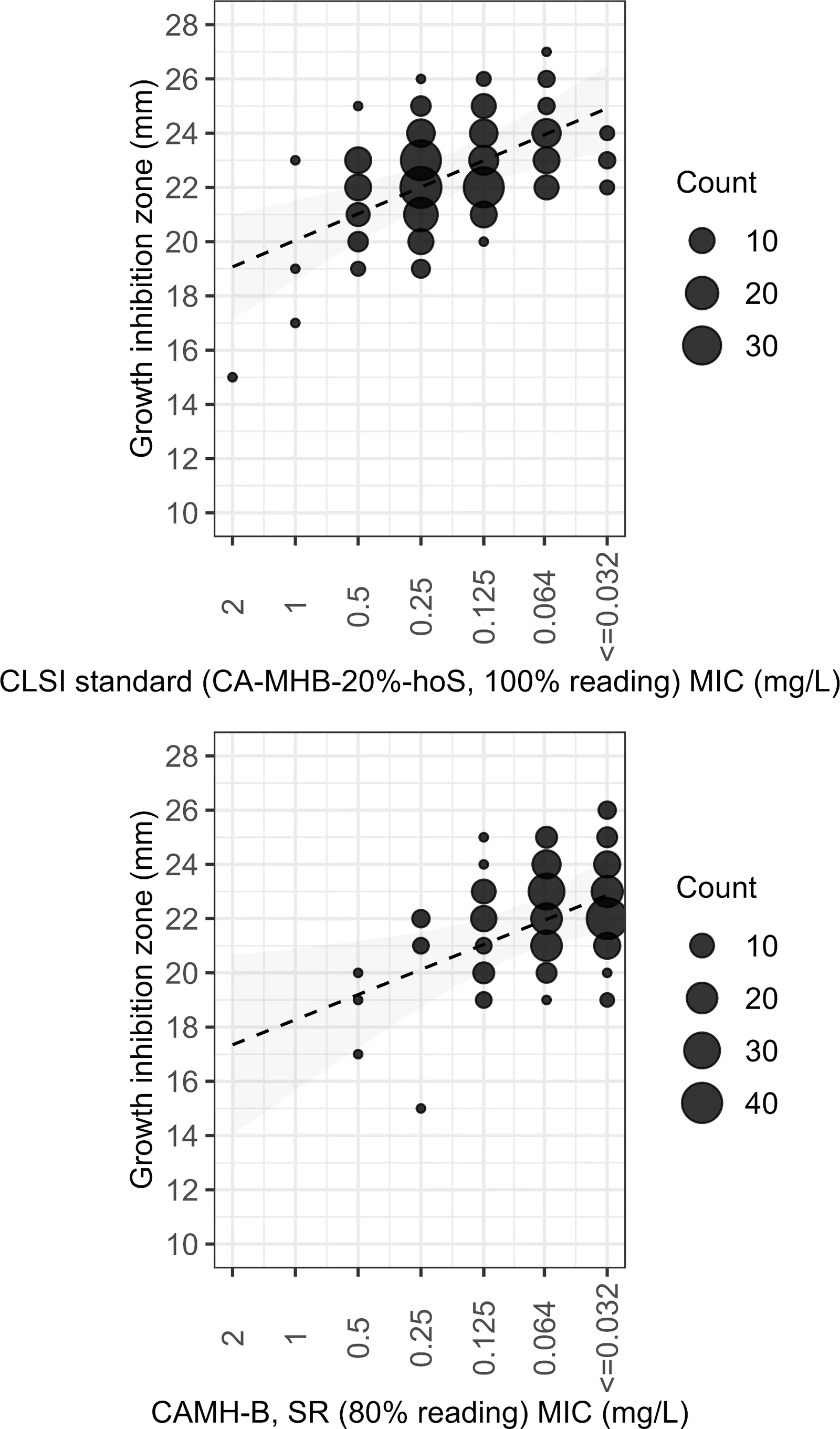
Correlation between zosurabalpin MICs determined using the CLSI and EUCAST methods and zosurabalpin growth inhibition zones. The black dashed line indicates the regression line.

The CLSI standard BMD and a CA-MHB-based method without heat-inactivated hoS applying a substantial reduction (SR) endpoint, defined as 80% growth inhibition (CA-MHB, SR), were compared with each other and with results obtained by DD using zosurabalpin-containing discs (5 µg) manufactured by Liofilchem and supplied by Roche (Fig. 5 and 6). MIC distributions obtained with standard CA-MHB and the SR endpoint were shifted toward lower values, with an average difference of approximately 1.9 doubling dilutions compared with the CLSI reference method (range between ≤0.008 and 0.5 mg/L, mean MICs: 0.063 µg/mL vs 0.293 µg/mL). MIC_50/90_ values were 0.25/0.5 µg/mL for the CLSI reference method and 0.0625/0.125 µg/mL for the CA-MHB, SR method. Of note, three isolates exhibiting an MIC of 1 mg/L by the CLSI reference method showed substantial variability when tested using the CA-MHB, SR (0.064, 0.125, and 0.5 mg/L), whereas the isolate with an MIC of 2 mg/L yielded an MIC of 0.25 mg/L by the CA-MHB, SR method.

DD results showed a strong inverse correlation with MICs obtained by both methods (Pearson *r* = −0.84/−0.81; Spearman *ρ* = −0.87/−0.86), indicating that DD represents a reliable phenotypic surrogate for MIC testing. The great majority of inhibition zone diameters clustered between 19 and 27 mm. A zone diameter of 19 mm may, therefore, be considered a tentative proxy for the ECOFF. Only two isolates, exhibiting elevated MICs of 1 and 2 mg/L by the CLSI reference method, showed clearly reduced inhibition zones of 17 and 15 mm, respectively. The remaining two isolates with MICs of 1 mg/L displayed inhibition zones of 19 and 23 mm, respectively.

## DISCUSSION

In this study, zosurabalpin demonstrated potent *in vitro* activity against a geographically and genomically diverse collection of 304 CRAB isolates, underscoring its potential as a future treatment option across heterogeneous global resistance landscapes. Consistent with previous reports, no evidence of plasmid-mediated resistance mechanisms was observed. Although resistance could theoretically arise through mutations, these may occur at a very low frequency (≤1 × 10⁻⁸ at exposures ≤4 × MIC), typically resulting in a high fold-change in MIC (10). Although pre-existing resistance to zosurabalpin appears uncommon, treatment-emergent resistance remains a realistic concern and should be closely monitored during clinical development and future clinical use. In contrast, mutations that activate the AdeABC efflux pump are associated only with modest increases in MIC values (10). Although the relevance of this finding remains uncertain, it is noteworthy that efflux pump activation has previously been implicated in reduced susceptibility to zosurabalpin, which is consistent with our findings. Importantly, no mutations in the known zosurabalpin targets (*lptF*, *lptG*, or *lpxM*) associated with elevated MICs were observed in the present study. Zosurabalpin directly targets the LptB₂FGC complex, thereby inhibiting LPS transport across the inner membrane. However, mutations in *lptD*, which encodes the outer-membrane LPS translocon, may indirectly influence susceptibility by altering LPS flux and outer-membrane biogenesis, potentially modulating the cellular dependence on inner-membrane LPS transport. Further studies are, therefore, required to elucidate the contribution of *lptD*-associated and efflux-related mechanisms to reduced susceptibility or resistance to zosurabalpin.

Consistent with our findings, the analysis of over 8,850 public *A. baumannii* genomes from clinical isolates identified 117 distinct protein-altering variants in *lptF*, *lptG*, and *lpxM*, corresponding to 749 variant-carrier observations. However, their functional relevance requires phenotypic validation, as most of these alterations likely represent natural polymorphism without an impact on zosurabalpin susceptibility (Table S2). Truncations resulting in premature stop codons were exceptionally rare, detected in only three cases for LptM and one case for LptG. Notably, none of the specific resistance mutations previously identified by Zampaloni et al.—nor any alternative substitutions at those exact amino acid positions—were detected in the data set. However, nine mutations causing amino acid substitutions near the sites described by Zampaloni et al. (within three amino acids) were identified across 38 strains. The relevance of these neighboring substitutions to zosurabalpin susceptibility requires experimental validation. Overall, based on the genomic data available from public repositories, these findings suggest a very low natural prevalence of strains harboring determinants associated with reduced zosurabalpin susceptibility.

EUCAST and CLSI recently released a joint document addressing modifications of AST methods, emphasizing that standard reference methods should be used for AST of novel antimicrobial compounds, and deviations should be considered exceptional only to ensure reliable *in vitro*–*in vivo* correlation (https://clsi.org/media/i54hohjl/modification-of-antimicrobial-susceptibility-testing-methods.pdf). Since modifying reference AST methods can create long-term barriers to test availability, as certain testing conditions may be difficult to reproduce at a large scale and require thorough verification, this practice should be avoided whenever possible. For zosurabalpin, CLSI has approved the use of CA-MHB supplemented with heat-inactivated 20% hoS for MIC determination due to aberrant readings (trailing, multiple skipped wells) when using the standard method with CA-MHB. To minimize deviations from the standard reference method, Roche has also reported MIC determination using standard CA-MHB with a substantial-reduction endpoint (SR; 80% growth inhibition) as a reliable approach that correlates well with the CLSI method (23). In the present study, MICs determined using the CA-MHB, SR method were consistently lower but showed substantial overall correlation with the CLSI reference broth microdilution method. However, isolates with borderline MICs (1–2 mg/L) as determined by the CLSI method did not consistently correlate with results obtained using the CA-MHB, SR approach. DD testing performed on standard Mueller–Hinton agar also appeared robust and showed good correlation with the reference BMD method. However, the data set contained only a limited number of isolates with MICs above the putative wt-population. Therefore, further studies including strains with reduced susceptibility are required to confirm the robustness of the observed correlations and to refine DD interpretive criteria.

Like colistin and rifabutin, zosurabalpin exhibited a carbapenemase-independent MIC distribution, supporting its potential role as a valuable therapeutic option when other agents, such as β-lactams or novel β-lactam/β-lactamase inhibitor combinations, are no longer effective or available.

Overall, zosurabalpin exhibits potent *in vitro* activity against CRAB, including NDM-producing CRAB isolates. In this subgroup, where therapeutic options remain limited, sulbactam-durlobactam is ineffective, and cefiderocol often shows reduced susceptibility. The CLSI-based broth microdilution method supplemented with heat-inactivated horse serum provided the most robust and reproducible susceptibility testing results and may serve as the preferred reference method for future susceptibility testing and breakpoint development.

## AFTER EPUB

*[This article was published on 16 September 2026 with an error in the byline. The byline was updated in the current version, posted on 21 September 2026.]*

## SUPPLEMENTAL MATERIAL

Supplemental material aac.00727-26-s0001.docx Fig. S1; Tables S1 and S2.

ASM does not own the copyrights to Supplemental Material that may be linked to, or accessed through, an article. The authors have granted ASM a non-exclusive, world-wide license to publish the Supplemental Material files. Please contact the corresponding author directly for reuse.
