# Supplementary material for: In vitro activity of zosurabalpin against a global set of carbapenem-resistant-A. baumannii clinical isolates
Source: Antimicrob Agents Chemother. 2026 Sep 16;70(10):e00727-26. doi: 10.1128/aac.00727-26 (PMC13689056; doi:10.1128/aac.00727-26)
Supplement: Supplementary file 1 [file aac.00727-26-s0001.docx]

**Supplementary data**

**Figure S1. Quality control.** Cefiderocol, colistin, sulbactam-durlobactam and zosurabalpin MIC values obtained using the BMD method. Replica numbers are on the x-axis and MICs on the y-axis. Green and yellow areas indicate the target and QC ranges: for *P. aeruginosa* ATCC 27853 and cefiderocol and colistin (EUCAST), and for *A. baumannii* NCTC 13304 and sulbactam–durlobactam (CLSI) and zosurabalpin (Roche).

**Table S1. Genetic and phenotypic features of the CRAB isolates used in this study**

| **Study CRAB No.** | **Country of isolation** | **Year of isolation** | **Original ID number** | **ST Pasteur** | **Carbapenemase** | **RMTase** | **Colistin resistance markers** | **Zosurabalpin resistance genes** | **Sulbactam Durlobactam resistance genes** | **Colistin** | **Cefiderocol MIC** | **Sulbactam-Durlobactam MIC** | **Rifabutin MIC** | **Zosurabalpin MIC (standard)** |
| --- | --- | --- | --- | --- | --- | --- | --- | --- | --- | --- | --- | --- | --- | --- |
| 1 | Switzerland | 2013 | Ab10 | 1 | blaOXA-72 |  |  |  |  | 0.5 | 0.25 | 0.5 | 0.016 | 0.125 |
| 2 | Switzerland | 2013 | Ab17 | 2 | blaOXA-23 |  |  | AdeS† (aa126),  lptD Δ692–704 |  | 0.5 | 0.25 | 1 | 0.016 | 2 |
| 3 | Switzerland | 2014 | Ab09 |  | blaOXA-72 |  |  |  |  | 0.5 | 0.25 | 1 | 0.016 | 0.25 |
| 4 | Switzerland | 2014 | Ab06 |  | blaOXA-23 | armA |  |  | ftsI_A515V | 0.25 | 0.125 | 4 | 0.016 | 0.25 |
| 5 | Switzerland | 2014 | Ab01 | 2 | blaOXA-23 | armA |  |  | ftsI_A515V | 0.25 | 0.25 | 4 | 0.016 | 0.25 |
| 6 | Switzerland | 2014 | Ab16 | 1 | blaOXA-23 |  |  |  |  | 0.5 | 0.5 | 1 | 0.016 | 0.25 |
| 7 | Switzerland | 2014 | Ab14 | 2 | blaOXA-23 |  |  |  |  | 0.5 | 2 | 4 | 0.016 | 0.25 |
| 8 | Switzerland | 2014 | Ab12 | 2 | blaOXA-72 |  |  |  |  | 0.25 | 0.125 | 1 | 0.016 | 0.25 |
| 9 | Switzerland | 2014 | Ab11 | 636 | blaOXA-72 |  |  |  |  | 0.5 | 0.5 | 1 | 0.016 | 0.5 |
| 10 | Switzerland | 2014 | Ab02 | 2 | blaOXA-23 | armA |  |  | ftsI_A515V | 0.125 | 0.125 | 8 | 0.016 | 0.0625 |
| 11 | Switzerland | 2014 | Ab13 | 1 | blaOXA-72 |  |  |  |  | 0.5 | 0.5 | 0.5 | 0.5 | 0.5 |
| 12 | Switzerland | 2014 | Ab03 | 2 | blaOXA-23 | armA |  |  | ftsI_A515V | 0.25 | 0.25 | 4 | 0.016 | 0.25 |
| 13 | Switzerland | 2014 | Ab04 | 2 | blaOXA-23 | armA |  |  | ftsI_A515V | 0.5 | 0.5 | 4 | 0.016 | 0.25 |
| 14 | Switzerland | 2014 | Ab05 | 2 | blaOXA-23 | armA |  |  | ftsI_A515V | 0.25 | 0.125 | 2 | 0.016 | 0.125 |
| 15 | Switzerland | 2014 | Ab08 |  | blaOXA-23 |  | pmrC_R125P |  |  | 4 | 0.5 | 4 | 0.016 | 0.25 |
| 16 | Switzerland | 2014 | Ab15 | 2 | - |  |  |  |  | 0.25 | 0.5 | 2 | 0.016 | 0.5 |
| 17 | Switzerland | 2016 | Ab47 | 2 | blaOXA-23 | armA |  |  | ftsI_A515V | 0.25 | 1 | 1 | 0.016 | 0.0625 |
| 18 | Switzerland | 2016 | Ab60 | 2 | blaOXA-23 | armA |  |  | ftsI_A515V | 0.25 | 0.25 | 4 | 0.016 | 0.125 |
| 19 | Switzerland | 2016 | Ab62 | 2 | blaOXA-23 | armA |  |  | ftsI_A515V | 0.5 | 1 | 1 | 0.016 | 0.0625 |
| 20 | Switzerland | 2016 | Ab61 | 2 | blaOXA-23 |  | prmA_A80V, prmB_T138A |  |  | 64 | 2 | 2 | 0.016 | 1 |
| 21 | Switzerland | 2016 | Ab57 | 575 | blaOXA-23 |  |  |  |  | 0.5 | 32 | 1 | 4 | 0.25 |
| 22 | Switzerland | 2017 | Ab56 | 25 | blaOXA-23 | armA |  |  |  | 0.5 | 8 | 1 | 16 | 0.25 |
| 23 | Switzerland | 2017 | Ab54 | 2 | blaOXA-23 |  |  |  | ftsI_A515V | 0.5 | 0.25 | 2 | 0.016 | 0.125 |
| 24 | Switzerland | 2018 | Ab18 | 25 | blaNDM-1 |  |  |  |  | 0.25 | 1 | 2 | 0.5 | 0.5 |
| 25 | Switzerland | 2018 | Ab20 | 1 | blaNDM-1 / blaOXA-23 |  |  |  |  | 0.5 | 1 | 4 | 0.016 | 0.0625 |
| 26 | Switzerland | 2018 | Ab21 | 107 | blaOXA-40 |  |  | lpxM_H104D,  lptD_T65A, lptD_I384N, lptD_L496V, lptD_K542E, lptD_D764N |  | 0.25 | 0.5 | 2 | 0.016 | 0.03125 |
| 27 | Switzerland | 2018 | Ab22 | 1 | blaOXA-23 |  |  |  |  | 0.5 | 4 | 0.5 | 0.016 | 0.0625 |
| 28 | Switzerland | 2018 | Ab23 | 20 | blaOXA-23 |  |  |  |  | 0.25 | 0.25 | 1 | 0.016 | 0.125 |
| 29 | Switzerland | 2018 | Ab24 | 1 | blaOXA-23 |  |  |  |  | 0.25 | 0.5 | 1 | 0.016 |  |
| 30 | Switzerland | 2018 | Ab25 | 1 | blaOXA-23 |  |  |  |  | 0.25 | 4 | 1 | 0.016 | 0.125 |
| 31 | Switzerland | 2018 | Ab26 | 108 | - |  |  |  |  |  | 0.125 | 1 | 0.016 | 0.25 |
| 32 | Switzerland | 2018 | Ab27 | 25 | blaOXA-58 |  |  |  |  | 0.25 | 0.03125 | 0.5 | 2 | 0.0625 |
| 33 | Switzerland | 2018 | Ab28 | 113 | - |  |  |  |  | 0.5 |  | 0.5 | 2 | 0.25 |
| 34 | Switzerland | 2018 | Ab29 | 113 | blaGES-14 |  |  | lptD_G21S, lptD_T65A, lptD_D356N, lptD_E421Q, lptD_V425A, lptD_N426D, lptD_D465N, lptD_L496V, lptD_Del509-510, lptD_A511T, lptD_S512Q, lptD_Q513L, lptD_G514N, lptD_L515N, lptD_D516P, lptD_InsG@678, lptD_I637F, lptD_N642K, lptD_V647I, lptD_L678V, lptD_S767T, lptD_P768S |  | 0.25 | 0.25 | 0.25 | 2 | 0.25 |
| 35 | Switzerland | 2018 | Ab30 | 47 | blaOXA-58 |  |  |  |  | 0.25 | 2 | 2 | 0.016 | 0.25 |
| 36 | Switzerland | 2018 | Ab31 | 3 | - |  |  |  |  |  | 0.125 | 2 | 0.016 | 0.25 |
| 37 | Switzerland | 2018 | Ab32 | 15 | blaOXA-58 |  |  | lptF_R5Q, lptG_T201S,  lptD_T65A, lptD_I384N, lptD_L496V |  | 0.25 |  | 1 | 0.016 | 0.25 |
| 38 | Switzerland | 2018 | Ab33 | 2 | - |  |  |  |  | 0.25 | 0.125 | 0.5 | 2 | 0.0625 |
| 39 | Switzerland | 2018 | Ab63 | 2 | blaOXA-23 | armA |  |  | ftsI_A515V | 0.25 | 0.25 | 2 | 0.016 | 0.125 |
| 40 | Switzerland | 2018 | Ab53 | 164 | blaOXA-23 / blaOXA-58 |  |  |  |  | 0.25 | 1 | 1 | 0.016 | 0.125 |
| 41 | Switzerland | 2019 | Ab36 | 2 | blaOXA-23 | armA |  |  | ftsI_A515V | 0.5 | 0.25 | 1 | 0.016 | 0.125 |
| 42 | Switzerland | 2019 | Ab51 | 2549 | blaOXA-72 |  |  |  |  | 0.25 | 0.25 | 2 | 0.5 | 0.5 |
| 43 | Switzerland | 2019 | Ab52 | 85 | blaNDM-1 |  |  |  |  | 0.25 | 4 | 8 | 0.016 | 0.125 |
| 44 | Switzerland | 2019 | Ab35 | 78 | blaOXA-72 |  |  |  |  | 0.25 | 0.0625 | 1 | 0.5 | 0.125 |
| 45 | Switzerland | 2019 | Ab34 | 636 | blaOXA-72 | armA |  |  |  | 0.25 | 0.5 | 1 | 0.016 | 0.125 |
| 46 | Switzerland | 2020 | Ab48 | 2 | blaOXA-23 | armA |  |  | ftsI_A515V | 0.25 | 0.25 | 1 | 0.016 | 0.125 |
| 47 | Switzerland | 2020 | Ab49 | 78 | blaOXA-72 |  |  |  |  | 0.25 | 0.5 | 1 | 0.016 | 0.125 |
| 48 | Switzerland | 2020 | Ab38 | 25 | blaOXA-23 | armA |  |  |  | 0.5 |  | 1 | 16 | 0.25 |
| 49 | Switzerland | 2020 | Ab50 | 164 | blaOXA-23 |  |  |  |  | 0.25 | 1 | 2 | 4 | 0.125 |
| 50 | Switzerland | 2020 | Ab58 | 2 | blaOXA-23 | armA |  |  | ftsI_A515V | 0.5 | 1 | 2 | 0.016 | 0.25 |
| 51 | Switzerland | 2020 | Ab39 | 2 | blaOXA-23 | armA | pmrB_T138A, pmrB_P170S, *eptA*::IS*Aba1* (codon 71) | - | ftsI_A515V | 16 | 0.25 | 2 | 0.016 | 0.25 |
| 52 | Switzerland | 2020 | Ab59 | 2 | blaOXA-23 |  |  |  | ftsI_A515V | 0.25 | 0.25 | 4 | 0.016 | 0.0625 |
| 53 | Switzerland | 2021 | Ab44 | 492 | blaNDM-1 / blaOXA-72 | armA |  |  |  | 0.25 | 8 | 4 | 0.016 | 0.0625 |
| 54 | Switzerland | 2021 | Ab71 | 2 | blaOXA-23 | armA |  |  | ftsI_A515V | 0.25 | 0.25 | 2 | 0.016 | 0.0625 |
| 55 | Switzerland | 2021 | Ab40 | 2 | blaOXA-23 | armA |  |  | ftsI_A515V | 0.25 | 0.5 | 4 | 0.016 | 0.0625 |
| 56 | Switzerland | 2021 | Ab43 | 2 | blaOXA-23 | armA |  |  | ftsI_A515V | 0.25 | 1 | 1 | 16 | 0.0625 |
| 57 | Switzerland | 2021 | Ab46 | 2 | blaNDM-1 / blaOXA-23 | armA |  |  |  | 0.25 | 4 | 64 | 0.016 | 0.0625 |
| 58 | Switzerland | 2021 | Ab41 | 25 | blaOXA-23 | armA |  |  |  | 0.5 | 0.25 | 0.5 | 16 | 0.125 |
| 59 | Switzerland | 2021 | Ab74 | 2 | blaOXA-23 |  |  |  |  | 0.25 | 0.5 | 2 | 0.016 | 0.25 |
| 60 | Switzerland | 2021 | Ab42 | 400 | - |  |  |  |  | 0.25 | 1 | 0.5 | 0.016 | 0.03125 |
| 61 | Switzerland | 2021 | Ab45 | 45 | blaOXA-72 |  |  |  |  | 0.25 | 8 | 1 | 0.016 | 0.125 |
| 62 | Switzerland | 2021 | Ab66 | 592 | blaOXA-72 | armA |  |  | ftsI_T526S | 0.125 | 0.5 | 32 | 0.5 | 0.0625 |
| 63 | Switzerland | 2021 | Ab69 | 1902 | blaOXA-23 | armA |  |  |  | 0.5 | 16 | 1 |  | 0.0625 |
| 64 | Switzerland | 2021 | Ab72 | 2 | blaOXA-23 | armA |  |  | ftsI_A515V | 0.25 | 0.25 | 2 | 0.016 | 0.125 |
| 65 | Switzerland | 2021 | Ab64 | 2 | blaOXA-23 | armA |  |  | ftsI_A515V | 0.25 | 0.25 | 4 | 0.016 | 0.0625 |
| 66 | Switzerland | 2021 | Ab67 | 164 | blaOXA-23 |  |  |  |  | 0.25 | 2 | 1 | 4 | 0.0625 |
| 67 | Switzerland | 2021 | Ab68 | 164 | blaOXA-23 |  |  |  |  | 2 | 0.5 | 2 | 0.016 |  |
| 68 | Switzerland | 2021 | Ab73 | 2 | blaOXA-23 | armA |  |  |  | 0.25 | 0.125 | 4 | 0.016 | 0.03125 |
| 69 | Switzerland | 2021 | Ab75 | 2 | blaOXA-23 | armA |  |  | ftsI_A515V | 0.25 | 0.25 | 2 | 0.016 | 0.0625 |
| 70 | Switzerland | 2021 | Ab77 | 641 | blaOXA-23 | armA |  |  |  | 0.25 | 0.5 | 2 | 2 | 0.125 |
| 71 | Switzerland | 2021 | Ab65 | 2 | blaOXA-23 | armA |  |  | ftsI_A515V | 0.25 | 0.25 | 2 | 0.016 | 0.0625 |
| 72 | Switzerland | 2021 | Ab70 | 2 | blaOXA-23 | armA |  |  | ftsI_A515V | 0.25 | 0.125 | 2 | 0.016 | 0.0625 |
| 73 | Switzerland | 2021 | Ab84 | 2 | blaOXA-23 | armA |  |  | ftsI_A515V | 0.5 | 0.5 | 4 | 16 | 0.25 |
| 74 | Switzerland | 2021 | Ab76 | 2 | blaOXA-23 | armA |  |  | ftsI_A515V | 0.25 | 0.125 | 4 | 0.016 | 0.0625 |
| 75 | Switzerland | 2022 | Ab83 | 2 | blaOXA-23 | armA |  |  | ftsI_A515V | 0.5 | 0.5 | 4 | 0.016 | 0.25 |
| 76 | Switzerland | 2022 | Ab96 | 2 | blaOXA-23 | armA |  |  | ftsI_A515V | 0.5 | 0.125 | 4 | 0.016 | 0.125 |
| 77 | Switzerland | 2022 | Ab109 | 2 | blaOXA-23 | armA |  |  |  | 0.25 | 8 | 1 | 1 | 0.125 |
| 78 | Switzerland | 2022 | Ab78 | 25 | blaOXA-23 | armA |  |  |  |  | 8 | 0.5 | 16 | 0.125 |
| 79 | Switzerland | 2022 | Ab79 | 2 | blaOXA-23 | armA |  |  | ftsI_A515V | 0.125 | 0.25 | 1 | 0.016 | 0.125 |
| 80 | Switzerland | 2022 | Ab80 | 492 | blaNDM-1 / blaOXA-72 | armA |  |  |  | 0.125 | 2 | 4 | 0.016 | 0.25 |
| 81 | Switzerland | 2022 | Ab81 | 78 | blaOXA-72 | armA |  |  |  | 0.125 | 0.25 | 4 | 0.016 | 0.125 |
| 82 | Switzerland | 2022 | Ab82 | 641 | blaOXA-23 | armA |  |  |  | 0.5 | 1 | 2 | 2 | 0.25 |
| 83 | Switzerland | 2022 | Ab85 | 2 | blaOXA-23 |  | pmrB_K263C, pmrB_T138A |  |  | 64 | 2 | 1 | 0.016 | 0.5 |
| 84 | Switzerland | 2022 | Ab86 | 2 | blaOXA-23 | armA |  |  | ftsI_A515V | 0.5 | 0.5 | 4 | 0.016 | 0.25 |
| 85 | Switzerland | 2022 | Ab87 | 2 | blaOXA-23 | armA |  |  | ftsI_A515V | 0.5 | 0.25 | 2 | 0.016 | 0.25 |
| 86 | Switzerland | 2022 | Ab88 | 2 | blaOXA-23 | armA |  |  | ftsI_A515V | 0.25 | 0.25 | 2 | 0.016 | 1 |
| 87 | Switzerland | 2022 | Ab89 | 2549 | blaOXA-72 | armA |  |  |  | 0.5 | 0.25 | 2 | 2 | 0.5 |
| 88 | Switzerland | 2022 | Ab90 | 164 | blaOXA-23 |  |  |  |  | 0.5 | 1 | 2 | 2 | 0.25 |
| 89 | Switzerland | 2022 | Ab91 | 2 | blaOXA-23 / blaOXA-72 | armA |  |  | ftsI_A515V | 0.5 | 0.5 | 2 | 0.016 | 0.25 |
| 90 | Switzerland | 2022 | Ab92 | 2 | blaOXA-23 / blaOXA-72 | armA |  |  | ftsI_A515V | 0.5 | 8 | 4 | 0.016 | 0.5 |
| 91 | Switzerland | 2022 | Ab93 | 2 | blaOXA-23 | armA |  |  | ftsI_A515V | 0.5 | 0.25 | 4 | 0.016 | 0.125 |
| 92 | Switzerland | 2022 | Ab94 | 492 | blaNDM-1 / blaOXA-72 | armA |  |  |  | 0.5 | 2 | 8 | 0.016 | 0.25 |
| 93 | Switzerland | 2022 | Ab95 | 164 | blaOXA-23 |  |  |  |  | 0.25 | 2 | 1 | 2 | 0.125 |
| 94 | Switzerland | 2022 | Ab97 | 2 | blaOXA-23 | armA |  |  | ftsI_A515V | 4 | 0.5 | 2 | 0.016 | 0.125 |
| 95 | Switzerland | 2022 | Ab98 | 2 | blaOXA-23 | armA |  |  | ftsI_A515V | 0.5 | 0.25 | 2 | 0.016 | 0.125 |
| 96 | Switzerland | 2022 | Ab99 | 2 | blaOXA-23 | armA |  |  | ftsI_A515V | 0.5 | 0.25 | 8 | 0.016 | 0.125 |
| 97 | Switzerland | 2022 | Ab100 | 2 | blaOXA-23 | armA |  |  | ftsI_A515V | 0.5 | 1 | 4 | 0.016 | 0.125 |
| 98 | Switzerland | 2022 | Ab101 | 78 | blaOXA-72 | armA |  |  |  | 0.5 | 2 | 2 | 0.016 | 0.25 |
| 99 | Switzerland | 2022 | Ab102 | 2 | blaOXA-23 | armA |  |  | ftsI_A515V | 1 | 1 | 4 | 0.016 | 0.25 |
| 100 | Switzerland | 2022 | Ab103 | 2 | blaOXA-23 |  |  |  | ftsI_A515V | 1 | 0.5 | 4 | 0.016 | 0.125 |
| 101 | Switzerland | 2022 | Ab104 | 2 | blaOXA-23 | armA |  |  | ftsI_A515V | 1 | 0.5 | 4 | 0.016 | 0.125 |
| 102 | Switzerland | 2022 | Ab105 | 78 | blaOXA-72 | armA |  |  |  | 1 | 0.25 | 0.5 | 0.016 | 0.125 |
| 103 | Switzerland | 2022 | Ab106 | 2 | blaOXA-23 | armA |  |  | ftsI_A515V | 0.25 | 0.25 | 2 | 0.016 | 0.125 |
| 104 | Switzerland | 2022 | Ab107 | 78 | blaOXA-72 |  |  |  |  | 0.25 | 0.125 | 4 | 0.016 | 0.25 |
| 105 | Switzerland | 2022 | Ab108 | 2 | blaOXA-23 | armA |  |  |  | 0.25 | 16 | 1 | 2 | 0.125 |
| 106 | Switzerland | 2022 | Ab110 | 2 | blaOXA-23 | armA | pmrB_R263C, eptA_R109H | lptD_S471R | ftsI_A515V | 32 | 0.5 | 2 | 0.016 | 0.25 |
| 107 | Switzerland | 2022 | Ab111 | 2 | blaOXA-23 | armA |  |  | ftsI_A515V | 0.25 | 1 | 4 | 0.016 | 0.25 |
| 108 | Switzerland | 2023 | Ab116 | 600 | blaNDM-1 / blaOXA-23 | armA |  |  | ftsI_A515V | 0.25 | 8 | 16 | 0.016 | 0.25 |
| 109 | Switzerland | 2023 | Ab137 | 2 | blaOXA-23 |  |  |  |  | 0.25 | 0.125 | 1 | 0.016 | 0.25 |
| 110 | Switzerland | 2023 | Ab138 | 2 | blaOXA-23 | armA |  |  | ftsI_A515V | 0.25 | 2 | 16 | 0.016 | 0.25 |
| 111 | Switzerland | 2023 | Ab141 | 2 | blaOXA-23 | armA |  |  | ftsI_A515V | 0.25 | 0.25 | 4 | 0.016 | 0.125 |
| 112 | Switzerland | 2023 | Ab112 | 85 | blaNDM-1 |  |  |  |  | 0.125 | 4 | 8 | 0.016 | 0.125 |
| 113 | Switzerland | 2023 | Ab55 | 78 | blaOXA-72 | armA |  |  |  | 0.25 | 0.25 | 2 | 0.03125 | 0.125 |
| 114 | Switzerland | 2023 | Ab113 | 164 | blaOXA-23 |  |  |  |  | 0.25 | 1 | 4 | 0.016 | 0.125 |
| 115 | Switzerland | 2023 | Ab114 | 85 | blaNDM-1 |  |  |  |  | 0.25 | 4 | 16 | 0.016 | 0.125 |
| 116 | Switzerland | 2023 | Ab124 | 2 | blaOXA-23 | armA |  |  |  | 0.5 | 16 | 2 | 2 | 0.25 |
| 117 | Switzerland | 2023 | Ab125 | 2 | blaOXA-23 | armA |  |  |  | 0.5 | 8 | 2 | 2 | 0.25 |
| 118 | Switzerland | 2023 | Ab122 | 2 | blaOXA-23 | armA |  |  |  | 1 | 8 | 2 | 1 | 0.25 |
| 119 | Switzerland | 2023 | Ab126 | 2 | blaOXA-23 | armA | pmrB_A227V |  | ftsI_A515V | 2 | 1 | 4 | 0.016 | 0.5 |
| 120 | Switzerland | 2023 | Ab129 | 2 | blaOXA-23 | armA |  |  |  | 0.5 | 16 | 1 | 2 | 0.25 |
| 121 | Switzerland | 2023 | Ab128 | 2 | blaOXA-23 | armA |  |  |  | 0.5 | 16 | 1 | 2 | 0.25 |
| 122 | Switzerland | 2023 | Ab127 | 2 | blaOXA-23 / blaOXA-72 |  |  |  |  | 1 | 0.25 | 1 | 0.016 | 0.5 |
| 123 | Switzerland | 2023 | Ab120 | 2 | blaOXA-23 | armA |  |  |  | 0.5 | 16 | 1 | 0.25 | 0.25 |
| 124 | Switzerland | 2023 | Ab132 | 19 | blaOXA-72 |  |  |  |  | 0.5 | 1 | 2 | 0.016 | 0.5 |
| 125 | Switzerland | 2023 | Ab130 | 400 | - |  |  |  |  | 0.5 | 0.5 | 1 | 0.016 | 0.25 |
| 126 | Switzerland | 2023 | Ab115 | 2 | blaOXA-23 | armA |  |  | ftsI_A515V | 0.25 | 0.125 | 2 | 0.016 | 0.5 |
| 127 | Switzerland | 2023 | Ab118 | 492 | blaOXA-72 | armA |  |  |  | 0.5 | 0.5 | 2 | 0.016 | 0.25 |
| 128 | Switzerland | 2023 | Ab119 | 2 | blaOXA-23 / blaOXA-72 | armA |  |  | ftsI_A515V | 0.5 | 0.125 | 4 | 0.016 | 0.25 |
| 129 | Switzerland | 2023 | Ab121 | 2 | blaNDM-1 / blaOXA-23 | armA |  |  |  | 2 | 8 | 32 | 0.016 | 0.25 |
| 130 | Switzerland | 2023 | Ab123 | 636 | blaOXA-72 | armA |  |  | ftsI_A515V | 0.5 | 2 | 2 | 0.016 | 0.25 |
| 131 | Switzerland | 2023 | Ab134 | 2 | blaOXA-23 | armA |  |  |  | 1 | 16 | 4 | 0.5 | 0.25 |
| 132 | Switzerland | 2023 | Ab136 | 2 | blaOXA-23 | armA |  |  | ftsI_A515V | 8 | 16 | 4 | 0.016 | 0.25 |
| 133 | Switzerland | 2023 | Ab139 | 2 | blaNDM-5 / blaOXA-23 | armA |  |  | ftsI_A515V | 0.25 | 1 | 4 | 0.016 | 0.25 |
| 134 | Switzerland | 2023 | Ab140 | 2 | blaOXA-23 | armA |  |  | ftsI_A515V | 0.5 | 0.25 | 4 | 0.016 | 0.125 |
| 135 | Switzerland | 2023 | Ab142 | 2 | blaNDM-5 / blaOXA-23 | armA |  |  | ftsI_A515V | 2 | 1 | 16 | 0.016 | 0.25 |
| 136 | Switzerland | 2023 | Ab143 | 2 | blaOXA-23 | armA |  |  | ftsI_A515V | 0.5 | 0.25 | 4 | 0.016 | 0.25 |
| 137 | Switzerland | 2023 | Ab163 | 2 | blaOXA-23 | armA |  |  | ftsI_A515V | 0.125 | 0.25 | 4 | 0.016 | 0.0625 |
| 138 | Switzerland | 2023 | Ab144 | 2 | blaOXA-23 | armA |  |  | ftsI_A515V | 0.25 | 0.25 | 4 | 0.016 | 0.25 |
| 139 | Switzerland | 2024 | Ab145 | 2 | blaOXA-23 | armA |  |  | ftsI_A515V | 0.125 | 0.125 | 4 | 2 | 0.0625 |
| 140 | Switzerland | 2024 | Ab150 | 2 | blaOXA-23 | armA |  |  | ftsI_A515V | 0.25 | 0.125 | 4 | 0.016 | 0.125 |
| 141 | Switzerland | 2024 | Ab153 | 2736 | blaOXA-23 |  |  |  | ftsI_A515V | 0.25 | 0.25 | 2 | 16 | 0.125 |
| 142 | Switzerland | 2024 | Ab158 | 2 | blaOXA-23 | armA |  |  |  | 0.5 | 16 | 0.5 | 1 | 0.25 |
| 143 | Switzerland | 2024 | Ab146 | 2 | blaOXA-23 | armA |  |  |  | 0.125 | 16 | 1 | 0.25 | 0.125 |
| 144 | Switzerland | 2024 | Ab147 | 23 | blaNDM-1 / blaOXA-58 |  |  |  |  | 0.125 | 2 | 1 | 2 | 0.25 |
| 145 | Switzerland | 2024 | Ab149 | 2 | blaOXA-23 | armA |  |  |  | 0.125 | 32 | 1 | 0.25 | 0.25 |
| 146 | Switzerland | 2024 | Ab151 | 2 | blaNDM-5 / blaOXA-23 | armA |  |  | ftsI_A515V | 0.25 | 4 | 32 | 0.016 | 0.25 |
| 147 | Switzerland | 2024 | Ab152 | 78 | blaOXA-72 |  |  |  |  | 0.25 | 0.0625 | 2 | 0.016 | 0.125 |
| 148 | Pakistan I | 2023 | Ab154 | 85 | blaOXA-23 |  |  |  | ftsI_T526S, pbpB1_S112P, pbpB1_A139V | 0.125 | 0.5 | 8 | 0.016 | 0.125 |
| 149 | Pakistan I | 2023 | Ab155 | 2 | blaOXA-23 |  |  |  |  | 0.25 | 8 | 2 | 0.016 | 0.125 |
| 150 | Pakistan I | 2023 | Ab156 | 85 | blaOXA-23 |  |  |  | ftsI_T526S, pbpB1_S112P, pbpB1_A139V | 0.25 | 1 | 16 | 0.016 | 0.125 |
| 151 | Pakistan I | 2023 | Ab157 | 2 | blaOXA-23 | armA |  |  | ftsI_A515V | 0.25 | 16 | 1 | 0.016 | 0.25 |
| 152 | Switzerland | 2024 | Ab159 | 2 | blaNDM-5 / blaOXA-23 |  |  | - | ftsI_A515V | 0.25 | 4 | 64 | 0.016 | 0.5 |
| 153 | Switzerland | 2024 | Ab160 | 2 | blaOXA-23 / blaOXA-72 |  |  |  |  | 0.25 | 0.125 | 0.5 | 0.016 | 0.25 |
| 154 | Switzerland | 2024 | Ab161 | 2 | blaOXA-23 | armA |  |  | ftsI_A515V | 0.5 | 1 | 2 | 0.016 | 0.25 |
| 155 | Switzerland | 2024 | Ab162 | 2 | blaNDM-1 / blaOXA-23 | armA |  |  |  | 0.5 | 2 | 8 | 0.5 | 0.125 |
| 156 | Pakistan | 2023 | Ab174 | 2 | blaOXA-23 |  |  |  |  | 0.25 | 8 | 4 | 0.016 | 0.0625 |
| 157 | Pakistan | 2023 | Ab175 | 85 | blaOXA-23 |  |  |  | ftsI_T526S, pbpB1_S112P, pbpB1_A139V | 0.125 | 0.5 | 16 | 0.016 | 0.03125 |
| 158 | Switzerland | 2024 | Ab167 | 492 | blaOXA-72 | armA |  |  |  | 0.25 | 0.5 | 4 | 0.016 | 0.0625 |
| 159 | Switzerland | 2024 | Ab165 | 2736 | blaOXA-23 | armA |  |  | ftsI_A515V | 0.25 | 0.25 | 1 | 4 | 0.03125 |
| 160 | Switzerland | 2024 | Ab166 | 2736 | blaOXA-23 | armA |  |  | ftsI_A515V | 0.25 | 0.25 | 8 | 4 | 0.0625 |
| 161 | Pakistan | 2023 | Ab170 | 2 | blaOXA-23 |  |  |  |  |  | 16 | 4 | 1 | 0.0625 |
| 162 | Switzerland | 2024 | Ab168 | 2 | blaOXA-23 | armA |  |  | ftsI_A515V | 0.125 | 0.25 | 4 | 0.016 | 0.03125 |
| 163 | Switzerland | 2024 | Ab169 | 2 | blaOXA-23 | armA |  |  | ftsI_A515V | 0.125 | 0.25 | 4 | 0.016 | 0.03125 |
| 164 | Israel | 2024 | 1 | 2 | blaOXA-72 |  |  |  |  | 1 | 2 | 4 | 2 | 0.5 |
| 165 | Israel | 2024 | 2 | 570 | blaNDM-1 / blaOXA-23 | armA |  |  | ftsI_A515V | 1 | 8 | 16 | 0.016 | 0.25 |
| 166 | Israel | 2024 | 3 | 2 | blaOXA-72 |  |  |  |  | 1 | 0.5 | 4 | 2 | 0.25 |
| 167 | Israel | 2024 | 4 | 2 | blaOXA-23 |  |  |  | ftsI_A515V | 1 | 1 | 2 | 0.016 | 0.125 |
| 168 | Israel | 2024 | 5 | 570 | blaNDM-1 / blaOXA-23 | armA |  |  | ftsI_A515V | 0.5 | 4 | 16 | 0.016 | 0.5 |
| 169 | Israel | 2024 | 6 | 3 | blaOXA-23 |  |  |  |  | 4 | 2 | 8 | 2 | 0.25 |
| 170 | Israel | 2023 | 7 | 2 | blaOXA-72 |  |  |  |  | 2 | 0.5 | 2 | 2 | 0.25 |
| 171 | Israel | 2023 | 8 | 2 | blaOXA-23 | armA |  |  | ftsI_A515V | 0.5 | 0.5 | 16 | 0.016 | 0.5 |
| 172 | Israel | 2023 | 9 | 570 | blaNDM-1 / blaOXA-23 | armA |  |  | ftsI_A515V | 0.5 | 16 | 16 | 0.016 | 0.5 |
| 173 | Israel | 2023 | 10 |  | blaNDM-1 / blaOXA-58 |  |  |  |  | 0.5 | 2 | 16 | 0.016 | 0.25 |
| 174 | Israel | 2023 | 11 | 2 | blaOXA-23 | armA |  |  | ftsI_A515V | 0.5 | 0.25 | 2 | 0.016 | 0.5 |
| 175 | Israel | 2024 | 12 | 2 | blaOXA-23 |  |  |  | ftsI_A515V | 1 | 0.5 | 2 | 0.016 | 0.0625 |
| 176 | Israel | 2024 | 13 | 2 | blaOXA-23 |  |  |  | ftsI_A515V | 1 | 1 | 2 | 0.016 | 0.125 |
| 177 | Israel | 2024 | 14 | 2 | blaOXA-23 |  |  |  | ftsI_A515V | 1 | 1 | 2 | 0.016 | 0.125 |
| 178 | Israel | 2024 | 15 |  | blaNDM-1 / blaOXA-58 |  |  |  |  | 0.5 | 4 | 16 | 0.016 | 0.25 |
| 179 | Israel | 2024 | 16 | 2 | blaNDM-1 / blaOXA-23 | armA |  |  |  | 1 | 2 | 64 | 0.016 | 0.25 |
| 180 | Israel | 2024 | 17 | 570 | blaNDM-1 / blaOXA-23 | armA |  |  | ftsI_A515V | 0.5 | 16 | 8 | 0.016 | 0.25 |
| 181 | Israel | 2023 | 18 | 570 | blaNDM-1 / blaOXA-23 | armA |  |  | ftsI_A515V | 0.5 | 4 | 8 | 0.016 | 0.25 |
| 182 | Israel | 2023 | 19 | 2 | blaOXA-23 |  |  |  |  | 1 | 0.25 | 1 | 2 | 0.125 |
| 183 | Israel | 2024 | 20 | 2 | blaOXA-23 |  |  |  | ftsI_A515V | 0.5 | 0.5 | 4 | 2 | 0.25 |
| 184 | Israel | 2023 | 21 | 2 | blaOXA-23 | armA | pmrB_P233S | lptD_R627H | ftsI_A515V | 0.5 | 1 | 4 | 0.016 | 0.25 |
| 185 | Israel | 2023 | 22 | 2 | blaOXA-23 | armA |  |  |  | 0.5 | 0.5 | 4 | 0.016 | 0.25 |
| 186 | Israel | 2023 | 23 | 2 | blaOXA-23 |  |  |  | ftsI_A515V | 0.5 | 1 | 4 | 0.016 | 0.125 |
| 187 | Israel | 2023 | 24 | 2 | blaOXA-72 |  |  |  |  | 64 | 0.5 | 1 | 0.5 | 0.5 |
| 188 | Israel | 2023 | 25 | 2 | blaOXA-23 | armA | pmrB_P233S |  | ftsI_A515V | 1 | 1 | 4 | 0.016 | 0.25 |
| 189 | Israel | 2023 | 26 | 2 | blaOXA-23 | armA |  |  | ftsI_A515V | 0.5 | 2 | 2 | 0.016 | 0.25 |
| 190 | Israel | 2023 | 27 | 2 | blaNDM-1 / blaOXA-23 | armA |  |  |  | 0.25 | 8 | 16 | 0.016 | 0.25 |
| 191 | Israel | 2023 | 28 | 2 | blaOXA-23 |  |  |  | ftsI_A515V | 1 | 1 | 2 | 0.016 | 0.0625 |
| 192 | Israel | 2023 | 29 | 2 | blaOXA-23 | armA |  |  | ftsI_A515V | 0.25 | 1 | 2 | 0.016 | 0.25 |
| 193 | Israel | 2023 | 30 | 2 | blaOXA-23 | armA |  |  | ftsI_A515V | 0.5 | 0.5 | 2 | 0.016 | 0.25 |
| 194 | Israel | 2023 | 31 | 3 | blaOXA-23 |  |  |  |  | 0.5 | 1 | 1 | 0.016 | 0.25 |
| 195 | Israel | 2023 | 32 |  | blaNDM-1 / blaOXA-58 |  |  |  |  | 0.5 | 2 | 16 | 0.016 | 0.25 |
| 196 | Israel | 2024 | 33 | 78 | blaOXA-72 | armA |  |  |  | 0.25 | 0.0625 | 1 | 0.016 | 0.125 |
| 197 | Israel | 2023 | 34 | 2 | blaNDM-1 / blaOXA-23 | armA |  |  |  | 1 | 4 | 16 | 0.016 | 0.25 |
| 198 | Israel | 2023 | 35 |  | blaNDM-1 / blaOXA-58 |  |  |  |  | 1 | 4 | 32 | 0.016 | 0.125 |
| 199 | Israel | 2023 | 36 | 2 | blaOXA-23 | armA |  |  | ftsI_A515V | 0.25 | 0.25 | 0.5 | 0.016 | 0.25 |
| 200 | Israel | 2023 | 37 | 2 | blaOXA-72 |  |  |  |  | 1 | 0.5 | 2 | 2 | 0.25 |
| 201 | Israel | 2023 | 38 | 3 | blaOXA-23 |  |  |  |  | 0.5 | 1 | 4 | 0.016 | 0.5 |
| 202 | Israel | 2023 | 39 | 2 | blaOXA-23 |  |  |  | ftsI_A515V | 1 | 1 | 2 | 0.016 | 0.125 |
| 203 | Israel | 2023 | 40 | 2 | blaOXA-23 | armA |  |  | ftsI_A515V | 1 | 0.25 | 2 | 0.016 | 0.25 |
| 204 | Israel | 2023 | 41 | 2 | blaOXA-23 |  |  |  | ftsI_A515V | 1 | 1 | 2 | 0.016 | 0.125 |
| 205 | Israel | 2023 | 42 | 2 | blaOXA-23 | armA |  |  | ftsI_A515V | 0.5 | 0.5 | 2 | 0.016 | 0.25 |
| 206 | Israel | 2023 | 43 | 2 | blaOXA-23 |  |  |  | ftsI_A515V | 0.5 | 1 | 2 | 0.016 | 0.125 |
| 207 | Israel | 2023 | 44 | 2 | blaOXA-72 |  |  |  |  | 1 | 1 | 2 | 2 | 0.5 |
| 208 | Israel | 2024 | 45 | 2 | blaNDM-1 / blaOXA-23 | armA |  |  |  | 0.5 | 8 | 8 | 0.016 | 0.25 |
| 209 | Israel | 2024 | 46 | 570 | blaOXA-23 | armA |  |  | ftsI_A515V | 0.5 | 1 | 4 | 0.016 | 0.25 |
| 210 | Israel | 2024 | 47 | 2 | blaOXA-23 |  |  |  | ftsI_A515V | 1 | 1 | 2 | 0.016 | 0.0625 |
| 211 | Israel | 2024 | 48 | 2 | blaOXA-23 |  |  |  | ftsI_A515V | 1 | 2 | 2 | 0.016 | 0.125 |
| 212 | Israel | 2023 | 49 | 2 | blaOXA-23 |  |  |  | ftsI_A515V | 0.5 | 1 | 4 | 0.016 | 0.125 |
| 213 | Pakistan II | 2024 | Ab176 | 2 | blaOXA-23 | armA |  |  |  | 0.25 | 0.5 | 4 | 0.016 | 0.25 |
| 214 | Switzerland | 2024 | Ab164 | 164 | blaOXA-23 |  |  |  |  | 0.03125 | 1 | 0.5 | 0.016 | 0.0625 |
| 215 | Switzerland | 2024 | Ab171 | 2 | blaOXA-23 | armA |  |  | ftsI_A515V | 0.25 | 0.125 | 1 | 0.016 | 0.0625 |
| 216 | Switzerland | 2024 | Ab172 | 164 | blaOXA-23 |  |  |  |  | 0.125 | 0.5 | 2 | 0.016 | 0.0625 |
| 217 | Pakistan I | 2023 | Ab173 | 2736 | blaOXA-23 | armA |  |  | ftsI_A515V | 0.125 | 0.25 | 4 | 2 | 0.0625 |
| 218 | Switzerland | 2025 | Ab178 | 85 | blaNDM-1 |  |  |  |  | 0.25 | 8 | 32 | 0.016 | 0.0625 |
| 219 | Türkiye |  | 1 | 2 | blaOXA-23 | armA |  |  | ftsI_A515V | 0.5 | 1 | 2 | 0.016 | 0.25 |
| 220 | Türkiye |  | 2 | 2 | blaOXA-23 | armA |  |  | ftsI_A515V | 0.5 | 0.25 | 2 | 0.016 | 0.125 |
| 221 | Türkiye |  | 3 | 2 | blaOXA-23 |  | pmrB_P233S |  | ftsI_A515V | 1 | 0.5 | 2 | 0.016 | 0.5 |
| 222 | Türkiye |  | 4 | 2 | blaOXA-23 | armA |  |  | ftsI_A515V | 0.5 | 0.5 | 4 | 0.016 | 0.125 |
| 223 | Türkiye |  | 5 | 2 | blaOXA-23 | armA |  |  | ftsI_A515V | 0.25 | 0.25 | 2 | 0.016 | 0.125 |
| 224 | Türkiye |  | 6 | 2 | blaOXA-23 | armA |  |  | ftsI_A515V | 0.25 | 0.25 | 2 | 0.016 | 0.5 |
| 225 | Türkiye |  | 7 | 2 | blaOXA-23 | armA |  |  | ftsI_A515V | 0.5 | 0.125 | 4 | 0.016 | 0.125 |
| 226 | Türkiye |  | 8 | 2 | blaOXA-23 | armA |  | AdeS† (aa126) |  | 0.5 | 8 | 1 | 16 | 1 |
| 227 | Türkiye |  | 9 | 2 | blaOXA-23 | armA |  |  | ftsI_A515V | 1 | 0.25 | 2 | 0.016 | 0.25 |
| 228 | Türkiye |  | 10 | 2 | blaOXA-23 | armA |  |  | ftsI_A515V | 0.5 | 0.25 | 4 | 0.016 | 0.25 |
| 229 | Türkiye |  | 11 | 2 | blaOXA-23 | armA |  |  |  | 0.5 | 4 | 1 | 16 | 0.25 |
| 230 | Türkiye |  | 12 | 2 | blaOXA-23 / blaOXA-72 | armA |  |  | ftsI_A515V | 0.25 | 0.25 | 2 | 0.016 | 0.125 |
| 231 | Türkiye |  | 13 | 2 | blaOXA-23 / blaOXA-72 | armA |  |  |  | 0.5 | 16 | 0.5 | 16 | 0.5 |
| 232 | Türkiye |  | 14 | 2 | blaOXA-23 / blaOXA-72 | armA |  |  | ftsI_A515V | 0.25 | 0.5 | 2 | 0.016 | 0.125 |
| 233 | Türkiye |  | 15 | 592 | blaNDM-1 / blaOXA-72 | rmtB |  |  | ftsI_T526S | 0.5 | 8 | 128 | 0.5 | 0.25 |
| 234 | Türkiye |  | 16 | 592 | blaNDM-1 / blaOXA-72 | rmtB |  |  | ftsI_T526S | 1 | 8 | 128 | 1 | 0.125 |
| 235 | Türkiye |  | 17 | 592 | blaNDM-1 / blaOXA-72 | rmtB |  |  | ftsI_T526S | 0.5 | 8 | 128 | 0.016 | 0.25 |
| 236 | Türkiye |  | 18 | 592 | blaNDM-1 / blaOXA-72 | rmtB |  |  | ftsI_T526S | 0.5 | 8 | 128 | 0.03125 | 0.125 |
| 237 | Türkiye |  | 19 | 592 | blaNDM-1 / blaOXA-72 | rmtB |  |  | ftsI_T526S | 0.5 | 4 | 128 | 0.5 | 0.25 |
| 238 | Türkiye |  | 20 | 592 | blaNDM-1 / blaOXA-72 | rmtB |  |  | ftsI_T526S | 2 | 4 | 128 | 2 | 0.25 |
| 239 | Türkiye |  | 21 | 2 | blaOXA-23 / blaOXA-72 | armA |  |  |  | 0.5 | 8 | 1 | 0.25 | 0.5 |
| 240 | Türkiye |  | 22 | 2 | blaOXA-23 / blaOXA-72 | armA | pmrB_T138A, pmrB_Δ207-211 |  |  | 64 | 4 | 1 | 16 | 0.5 |
| 241 | Türkiye |  | 23 | 2 | blaOXA-23 / blaOXA-72 | armA | pmrB_R263C, eptA_R109H |  | ftsI_A515V | 32 | 0.25 | 4 | 0.016 | 0.5 |
| 242 | Türkiye |  | 24 | 2 | blaOXA-23 / blaOXA-72 | armA |  |  | ftsI_A515V | 0.25 | 0.25 | 2 | 0.016 | 0.5 |
| 243 | Türkiye |  | 25 | 592 | blaNDM-1 / blaOXA-72 | rmtB | pmrB_T232I |  | ftsI_T526S | 4 | 8 | 128 | 0.5 | 0.25 |
| 244 | Türkiye |  | 26 | 592 | blaNDM-1 / blaOXA-72 | rmtB |  |  | ftsI_T526S | 0.25 | 2 | 128 | 0.25 | 0.25 |
| 245 | Türkiye |  | 27 | 2 | blaOXA-23 / blaOXA-72 | armA |  |  | ftsI_A515V | 0.25 | 0.25 | 2 | 0.016 | 0.25 |
| 246 | Türkiye |  | 28 | 164 | blaOXA-23 / blaOXA-72 |  |  |  |  | 0.25 | 2 | 1 | 1 | 0.25 |
| 247 | Türkiye |  | 29 | 2 | blaOXA-23 / blaOXA-72 | armA |  |  |  | 0.25 | 0.25 | 1 | 16 | 0.25 |
| 248 | Türkiye |  | 30 | 2 | blaOXA-23 / blaOXA-72 |  |  |  |  | 0.25 | 0.125 | 2 | 0.03125 | 0.5 |
| 249 | Türkiye |  | 31 | 2 | blaOXA-23 / blaOXA-72 | armA |  |  | ftsI_A515V | 0.25 | 0.5 | 2 | 0.016 | 0.5 |
| 250 | Türkiye |  | 32 | 2 | blaOXA-23 / blaOXA-72 | armA |  |  |  | 0.25 | 0.5 | 1 | 16 | 0.5 |
| 251 | Türkiye |  | 33 | 2 | blaOXA-23 / blaOXA-72 | armA |  |  |  | 0.25 | 0.25 | 1 | 16 | 0.5 |
| 252 | Türkiye |  | 34 | 78 | blaOXA-72 | armA |  |  |  | 0.5 | 0.0625 | 2 | 0.016 | 0.25 |
| 253 | Türkiye |  | 35 | 2 | blaOXA-23 | armA |  |  | ftsI_A515V | 0.5 | 0.25 | 2 | 0.016 | 0.125 |
| 254 | Türkiye |  | 36 | 78 | blaOXA-72 | armA |  |  |  | 0.25 | 0.0625 | 2 | 0.016 | 0.25 |
| 255 | Türkiye |  | 37 | 2 | blaOXA-23 / blaOXA-72 | armA |  |  |  | 0.5 | 0.25 | 1 | 16 | 0.25 |
| 256 | Türkiye |  | 38 | 78 | blaOXA-72 | armA | pmrB_R231L | lptG_T201I,  lptD_T65A, lptD_G222A, lptD_Ins 4aas 279, lptD_D281G, lptD_I284G, lptD_V290I, lptD_D309E, lptD_A312P, lptD_Q315K, lptD_Q329E, lptD_H350N, lptD_D356N, lptD_N368H, lptD_F376Y, lptD_S377A, lptD_I384N, lptD_E421D, lptD_D465G, lptD_V525I, lptD_D562E, lptD_V646I, lptD_H725L, lptD_D769N, lptD_S769N, lptD_S787S, lptD_L795I |  | 1 | 0.0625 | 2 | 0.125 | 0.5 |
| 257 | Türkiye |  | 39 | 2 | blaOXA-23 | armA | pmrB_A226T |  |  | 0.25 | 0.125 | 2 | 0.25 | 0.5 |
| 258 | Türkiye |  | 40 | 78 | blaOXA-72 | armA |  |  |  | 0.5 | 0.0625 | 2 | 0.016 | 0.25 |
| 259 | Türkiye |  | 41 | 78 | blaOXA-72 | armA |  |  |  | 0.5 | 0.0625 | 2 | 0.016 | 0.25 |
| 260 | Türkiye |  | 42 | 78 | blaOXA-72 | armA |  |  |  | 0.5 | 0.0625 | 4 | 0.016 | 0.25 |
| 261 | Türkiye |  | 43 | 2 | blaOXA-23 / blaOXA-72 | armA |  |  | ftsI_A515V | 0.5 | 0.5 | 4 | 0.016 | 0.5 |
| 262 | Türkiye |  | 44 |  | blaOXA-72 | armA |  |  |  | 0.5 | 0.25 | 4 | 2 | 0.125 |
| 263 | Türkiye |  | 45 | 2 | blaOXA-23 |  | pmrB_A226T |  |  | 0.5 | 0.25 | 1 | 0.016 | 0.125 |
| 264 | Türkiye |  | 46 | 592 | blaNDM-1 / blaOXA-72 | rmtB |  |  | ftsI_T526S | 0.25 | 4 | 128 | 0.125 | 0.25 |
| 265 | Türkiye |  | 47 | 2 | blaOXA-23 / blaOXA-72 | armA |  |  |  | 0.5 | 0.25 | 1 | 16 | 0.5 |
| 266 | Türkiye |  | 48 | 2 | blaOXA-23 / blaOXA-72 | armA |  |  |  | 0.5 | 0.25 | 4 | 16 | 0.25 |
| 267 | Türkiye |  | 49 | 78 | blaOXA-72 |  |  |  |  | 0.5 | 0.25 | 4 | 0.016 | 0.5 |
| 268 | Türkiye |  | 50 | 2 | blaOXA-23 / blaOXA-72 | armA |  |  |  | 0.5 | 0.125 | 1 | 16 | 0.0625 |
| 269 | Türkiye |  | 52 | 2 | blaOXA-23 / blaOXA-72 | armA |  |  |  | 0.5 | 0.25 | 1 | 16 | 0.0625 |
| 270 | Türkiye |  | 53 | 2 | blaOXA-23 / blaOXA-72 | armA |  |  |  | 0.5 | 8 | 1 | 0.25 | 0.125 |
| 271 | Türkiye |  | 54 | 592 | blaNDM-1 / blaOXA-72 | rmtB |  |  | ftsI_T526S | 0.25 | 4 | 128 | 0.016 | 0.25 |
| 272 | Türkiye |  | 55 | 592 | blaNDM-1 / blaOXA-72 | rmtB |  |  | ftsI_T526S | 0.25 | 8 | 128 | 0.03125 | 0.25 |
| 273 | Türkiye |  | 56 | 592 | blaNDM-1 / blaOXA-72 | rmtB |  |  | ftsI_T526S | 0.25 | 8 | 128 | 0.125 | 0.125 |
| 274 | Türkiye |  | 57 | 717 | blaOXA-23 / blaOXA-72 |  |  |  |  | 1 | 0.125 | 1 | 16 | 0.25 |
| 275 | Türkiye |  | 58 | 2 | blaOXA-23 / blaOXA-72 | armA |  |  | ftsI_A515V | 0.5 | 0.5 | 1 | 0.016 | 0.125 |
| 276 | Türkiye |  | 59 | 592 | blaNDM-1 / blaOXA-72 | rmtB |  |  | ftsI_T526S | 0.5 | 8 | 128 | 0.016 | 0.25 |
| 277 | Türkiye |  | 60 | 592 | blaNDM-1 / blaOXA-72 | rmtB |  |  | ftsI_T526S | 1 | 8 | 128 | 0.016 | 0.25 |
| 278 | Türkiye |  | 61 | 592 | blaNDM-1 / blaOXA-72 | rmtB |  |  | ftsI_T526S | 0.5 | 8 | 128 | 0.016 | 0.25 |
| 279 | Türkiye |  | 62 | 592 | blaNDM-1 / blaOXA-72 | rmtB |  |  | ftsI_T526S | 1 | 4 | 128 | 0.5 | 0.25 |
| 280 | Türkiye |  | 63 | 592 | blaNDM-1 / blaOXA-72 | rmtB |  |  | ftsI_T526S | 0.5 | 8 | 128 | 0.125 | 0.25 |
| 281 | Türkiye |  | 64 | 592 | blaNDM-1 / blaOXA-72 | rmtB |  |  | ftsI_T526S | 0.5 | 16 | 128 | 0.125 | 0.0625 |
| 282 | Switzerland | 2025 | Ab180 |  | blaNDM-1 | armA |  | - | ftsI_A515V | 0.25 | 4 | 4 | 0.016 | 0.125 |
| 283 | Switzerland | 2025 | Ab177 | 16 | blaOXA-23 / blaOXA-58 |  |  |  |  | 0.5 | 0.5 | 4 | 0.016 | 0.25 |
| 284 | Switzerland | 2025 | Ab181 | 492 | blaNDM-1 / blaOXA-72 | armA |  |  |  | 0.25 | 2 | 2 | 0.016 | 0.25 |
| 285 | Switzerland | 2025 | Ab182 | 2 | blaOXA-72 |  |  |  |  | 0.25 | 0.5 | 0.5 | 0.016 | 0.125 |
| 286 | Switzerland | 2025 | Ab183 | 492 | blaNDM-1 / blaOXA-72 | armA |  |  |  | 0.25 | 4 | 8 | 0.016 | 0.25 |
| 287 | Australia | 2012 | PW01b | 2 | blaOXA-23 |  |  |  |  | 0.125 | 0.125 | 1 | 0.016 | 0.25 |
| 288 | Australia | 2012 | WM00 | 187 | blaOXA-23 |  |  |  |  | 0.5 | 0.25 | 1 | 0.016 | 0.125 |
| 289 | Australia | 2012 | RB01 | 2 | blaOXA-23 |  |  |  |  | 0.125 | 0.125 | 0.5 | 0.016 | 0.125 |
| 290 | Australia | 2012 | RB02d1 | 2 | blaOXA-23 |  |  |  |  | 0.125 | 0.25 | 1 | 0.016 | 0.0625 |
| 291 | Pakistan | unknown | Pk.M9 | 2 | blaOXA-23 | armA |  |  | ftsI_A515V | 0.25 | 16 | 2 | 0.016 | 0.125 |
| 292 | Pakistan | unknown | Pk.M10 | 1 | blaOXA-23 |  |  |  |  | 0.25 | 0.125 | 1 | 0.016 | 0.125 |
| 293 | Pakistan | unknown | Pk.M11 | 1 | blaOXA-23 |  |  |  |  | 0.125 | 0.125 | 1 | 0.016 | 0.125 |
| 294 | Pakistan | unknown | Pk.A2 | 2 | blaOXA-23 | armA |  |  | ftsI_A515V | 0.125 | 64 | 4 | 16 | 0.125 |
| 295 | Pakistan | unknown | Pk.A3 | 570 | blaNDM-1 / blaOXA-23 | armA |  | - | ftsI_A515V | 0.125 | 2 | 8 | 0.016 | 0.125 |
| 296 | Pakistan | unknown | Pk.A8a | 570 | blaOXA-23 | armA |  |  | ftsI_A515V | 0.125 | 8 | 64 | 0.016 | 0.125 |
| 297 | Pakistan | unknown | Pk.A8b | 570 | blaOXA-23 | armA |  |  | ftsI_A515V | 0.125 | 8 | 32 | 0.016 | 0.0625 |
| 298 | Ethiopia | 2024 |  | 740 | blaNDM-9 |  |  | lptD_A49T, lptD_T65A, lptD_I384N, lptD_L496V |  | 0.25 | 16 | 16 | 16 | 0.0625 |
| 299 | Ethiopia | 2024 |  | 740 | blaNDM-9 |  |  |  |  | 0.125 | 16 | 16 | 16 | 0.125 |
| 300 | Ethiopia | 2024 |  | 2 | blaNDM-1 / blaOXA-23 | armA |  |  | ftsI_A515V | 0.125 | 8 | 8 | 16 | 0.5 |
| 301 | Ethiopia | 2024 |  | 2 | blaNDM-1 / blaOXA-23 | armA |  |  |  | 0.125 | 16 | 16 | 0.016 | 0.25 |
| 302 | Ethiopia | 2024 |  | 2 | blaNDM-1 / blaOXA-23 | armA |  |  |  | 0.25 | 8 | 16 | 0.016 | 0.25 |
| 303 | Ethiopia | 2024 |  | 2 | blaNDM-1 / blaOXA-23 | armA |  |  |  | 0.25 | 16 | 16 | 0.016 | 0.25 |
| 304 | Ethiopia | 2024 |  | 2 | blaNDM-1 / blaOXA-23 | armA |  |  |  | 0.25 | 4 | 32 | 0.016 | 0.25 |

**Table S2. Amino acid–altering variants in LptF, LptG, and LpxM among publicly available *A. baumannii* genomes and their overlap with zosurabalpin resistance-associated mutations reported by Zampaloni *et al.***

| **Gene** | **Aminoacid change** | **Aminoacid variation** | **Strains carrying mutations** | **Callable strains** | **Frequency (%)** | **Nearest Zampaloni’s site (fold increase in zosurabalpin MIC compared to the isogenic parental strain)** | **Distance from Zampaloni's site** |
| --- | --- | --- | --- | --- | --- | --- | --- |
| *lptF* | Ser252Pro | missense | 14 | 8877 | 0.158 | E249K (32–256×) | 3 aa |
| *lptF* | Thr321Ile | missense | 14 | 8877 | 0.158 | I319_K320N (>256×); K320N (64×); K320E (256×); K320T (16×); K320Q (32×); K320del (>256×); K320dup (256×); R322C (32–128×); R322G (8–16×); R322L (32–64×); R322S (16×) | 1 aa |
| *lptF* | Ser163Pro | missense | 7 | 8877 | 0.079 |  |  |
| *lptF* | Gly182Asp | missense | 4 | 8877 | 0.045 |  |  |
| *lptF* | Pro276Ser | missense | 4 | 8877 | 0.045 |  |  |
| *lptF* | Ser254Thr | missense | 4 | 8877 | 0.045 |  |  |
| *lptF* | Ala179Val | missense | 3 | 8877 | 0.034 |  |  |
| *lptF* | Ala159Glu | missense | 2 | 8877 | 0.023 |  |  |
| *lptF* | Phe128Ile | missense | 2 | 8877 | 0.023 |  |  |
| *lptF* | Ser94Asn | missense | 2 | 8877 | 0.023 |  |  |
| *lptF* | Val339Phe | missense | 2 | 8877 | 0.023 |  |  |
| *lptF* | Ala343Thr | missense | 1 | 8877 | 0.011 |  |  |
| *lptF* | Arg100Ser | missense | 1 | 8877 | 0.011 |  |  |
| *lptF* | Arg138Cys | missense | 1 | 8877 | 0.011 |  |  |
| *lptF* | Arg30Cys | missense | 1 | 8877 | 0.011 |  |  |
| *lptF* | Arg55His | missense | 1 | 8877 | 0.011 | E58G (8×); E58K (>256×) | 3 aa |
| *lptF* | Gly246Asp | missense | 1 | 8877 | 0.011 | E249K (32–256×) | 3 aa |
| *lptF* | Gly28Ala | missense | 1 | 8877 | 0.011 | L62H (64–256×) |  |
| *lptF* | Leu74Met | missense | 1 | 8877 | 0.011 | L36Q (32×) |  |
| *lptF* | Phe217Leu | missense | 1 | 8877 | 0.011 | K285N (4×) |  |
| *lptF* | Pro263Arg | missense | 1 | 8877 | 0.011 | K285N (4×) |  |
| *lptF* | Ser114Ala | missense | 1 | 8877 | 0.011 | W78stop (32×) |  |
| *lptF* | Ser254Leu | missense | 1 | 8877 | 0.011 |  |  |
| *lptF* | Ser286Phe | missense | 1 | 8877 | 0.011 |  |  |
| *lptF* | Thr61Pro | missense | 1 | 8877 | 0.011 | L62H (64–256×) | 1 aa |
| *lptF* | Val111Met | missense | 1 | 8877 | 0.011 | L36Q (32×) |  |
| *lptF* | Val196Ile | missense | 1 | 8877 | 0.011 | K285N (4×) |  |
| *lptG* | Thr201Ser | missense | 93 | 8883 | 1.047 | K285N (4×) |  |
| *lptG* | Thr201Ile | missense | 77 | 8883 | 0.867 | W78stop (32×) |  |
| *lptG* | Arg5Gln | missense | 17 | 8883 | 0.191 |  |  |
| *lptG* | Ser206Arg | missense | 14 | 8883 | 0.158 |  |  |
| *lptG* | Ile282Val | missense | 13 | 8883 | 0.146 |  |  |
| *lptG* | Phe194Ile | missense | 7 | 8883 | 0.079 |  |  |
| *lptG* | Glu65Asp | missense | 4 | 8883 | 0.045 |  |  |
| *lptG* | Ala142Val | missense | 2 | 8883 | 0.023 |  |  |
| *lptG* | Asn47Ser | missense | 2 | 8883 | 0.023 |  |  |
| *lptG* | Gly103Asp | missense | 2 | 8883 | 0.023 |  |  |
| *lptG* | Ser70fs | frameshift | 2 | 8883 | 0.023 |  |  |
| *lptG* | Ala163Val | missense | 1 | 8883 | 0.011 |  |  |
| *lptG* | Ala229Val | missense | 1 | 8883 | 0.011 |  |  |
| *lptG* | Ala330Val | missense | 1 | 8883 | 0.011 |  |  |
| *lptG* | Arg354Trp | missense | 1 | 8883 | 0.011 |  |  |
| *lptG* | Asp258Glu | missense | 1 | 8883 | 0.011 |  |  |
| *lptG* | Gly37Asp | missense | 1 | 8883 | 0.011 | L36Q (32×) | 1 aa |
| *lptG* | His137Tyr | missense | 1 | 8883 | 0.011 |  |  |
| *lptG* | Ile251Thr | missense | 1 | 8883 | 0.011 |  |  |
| *lptG* | Leu143Phe | missense | 1 | 8883 | 0.011 |  |  |
| *lptG* | Ser349Gly | missense | 1 | 8883 | 0.011 |  |  |
| *lptG* | Val114Ile | missense | 1 | 8883 | 0.011 |  |  |
| *lptG* | Val175Leu | missense | 1 | 8883 | 0.011 |  |  |
| *lptG* | Val238Met | missense | 1 | 8883 | 0.011 |  |  |
| *lptG* | Val337Leu | missense | 1 | 8883 | 0.011 |  |  |
| *lpxM* | Val326Leu | missense | 266 | 8850 | 3.006 |  |  |
| *lpxM* | Leu35Phe | missense | 17 | 8850 | 0.192 |  |  |
| *lpxM* | Val261Ile | missense | 17 | 8850 | 0.192 |  |  |
| *lpxM* | Arg99His | missense | 10 | 8850 | 0.113 |  |  |
| *lpxM* | Asp297Glu | missense | 10 | 8850 | 0.113 |  |  |
| *lpxM* | Leu32Ile | missense | 7 | 8850 | 0.079 |  |  |
| *lpxM* | Ser160Asn | missense | 7 | 8850 | 0.079 |  |  |
| *lpxM* | Thr169Ile | missense | 7 | 8850 | 0.079 |  |  |
| *lpxM* | Arg307Lys | missense | 5 | 8850 | 0.056 |  |  |
| *lpxM* | His104Asp | missense | 4 | 8850 | 0.045 |  |  |
| *lpxM* | Lys144Asn | missense | 4 | 8850 | 0.045 |  |  |
| *lpxM* | Lys282Asn | missense | 4 | 8850 | 0.045 | K285N (4×) | 3 aa |
| *lpxM* | Ala224Thr | missense | 3 | 8850 | 0.034 |  |  |
| *lpxM* | Lys303Ile | missense | 3 | 8850 | 0.034 |  |  |
| *lpxM* | Tyr58His | missense | 3 | 8850 | 0.034 |  |  |
| *lpxM* | Ala158Thr | missense | 2 | 8850 | 0.023 |  |  |
| *lpxM* | Asn130Asp | missense | 2 | 8850 | 0.023 |  |  |
| *lpxM* | Asn148Tyr | missense | 2 | 8850 | 0.023 |  |  |
| *lpxM* | Gln272Lys | missense | 2 | 8850 | 0.023 |  |  |
| *lpxM* | Gly25Val | missense | 2 | 8850 | 0.023 |  |  |
| *lpxM* | Pro321Leu | missense | 2 | 8850 | 0.023 |  |  |
| *lpxM* | Ser325Thr | missense | 2 | 8850 | 0.023 |  |  |
| *lpxM* | Thr137Ile | missense | 2 | 8850 | 0.023 |  |  |
| *lpxM* | Tyr271His | missense | 2 | 8850 | 0.023 |  |  |
| *lpxM* | Tyr277Cys | missense | 2 | 8850 | 0.023 |  |  |
| *lpxM* | Val146Phe | missense | 2 | 8850 | 0.023 |  |  |
| *lpxM* | Ala255Thr | missense | 1 | 8850 | 0.011 |  |  |
| *lpxM* | Ala33Val | missense | 1 | 8850 | 0.011 |  |  |
| *lpxM* | Asn196Asp | missense | 1 | 8850 | 0.011 |  |  |
| *lpxM* | Asn213Ile | missense | 1 | 8850 | 0.011 |  |  |
| *lpxM* | Asn287Thr | missense | 1 | 8850 | 0.011 | K285N (4×) | 2 aa |
| *lpxM* | Asn295Ile | missense | 1 | 8850 | 0.011 | W78stop (32×) |  |
| *lpxM* | Asp152Gly | missense | 1 | 8850 | 0.011 |  |  |
| *lpxM* | Asp257del | inframe deletion | 1 | 8850 | 0.011 |  |  |
| *lpxM* | Cys133Tyr | missense | 1 | 8850 | 0.011 |  |  |
| *lpxM* | Glu81Val | missense | 1 | 8850 | 0.011 | W78stop (32×) | 3 aa |
| *lpxM* | Gly173Ser | missense | 1 | 8850 | 0.011 |  |  |
| *lpxM* | Ile119Leu | missense | 1 | 8850 | 0.011 |  |  |
| *lpxM* | Ile177Phe | missense | 1 | 8850 | 0.011 |  |  |
| *lpxM* | Ile270Val | missense | 1 | 8850 | 0.011 |  |  |
| *lpxM* | Ile296Phe | missense | 1 | 8850 | 0.011 |  |  |
| *lpxM* | Leu190Met | missense | 1 | 8850 | 0.011 |  |  |
| *lpxM* | Leu214Ile | missense | 1 | 8850 | 0.011 |  |  |
| *lpxM* | Lys221Asn | missense | 1 | 8850 | 0.011 |  |  |
| *lpxM* | Lys40Thr | missense | 1 | 8850 | 0.011 |  |  |
| *lpxM* | Met139Ile | missense | 1 | 8850 | 0.011 |  |  |
| *lpxM* | Met1Ile | missense | 1 | 8850 | 0.011 |  |  |
| *lpxM* | Met241Leu | missense | 1 | 8850 | 0.011 |  |  |
| *lpxM* | Phe123Leu | missense | 1 | 8850 | 0.011 |  |  |
| *lpxM* | Ser160Ile | missense | 1 | 8850 | 0.011 |  |  |
| *lpxM* | Ser18Arg | missense | 1 | 8850 | 0.011 |  |  |
| *lpxM* | Ser212Asn | missense | 1 | 8850 | 0.011 |  |  |
| *lpxM* | Ser280Ile | missense | 1 | 8850 | 0.011 |  |  |
| *lpxM* | Thr125fs | frameshift | 1 | 8850 | 0.011 |  |  |
| *lpxM* | Thr137Arg | missense | 1 | 8850 | 0.011 |  |  |
| *lpxM* | Thr194Ile | missense | 1 | 8850 | 0.011 |  |  |
| *lpxM* | Thr194Pro | missense | 1 | 8850 | 0.011 |  |  |
| *lpxM* | Thr254Ile | missense | 1 | 8850 | 0.011 |  |  |
| *lpxM* | Thr299Ala | missense | 1 | 8850 | 0.011 |  |  |
| *lpxM* | Thr324Arg | missense | 1 | 8850 | 0.011 |  |  |
| *lpxM* | Thr38fs | frameshift | 1 | 8850 | 0.011 |  |  |
| *lpxM* | Trp86Leu | missense | 1 | 8850 | 0.011 |  |  |
| *lpxM* | Val120Leu | missense | 1 | 8850 | 0.011 |  |  |
| *lpxM* | Val155Met | missense | 1 | 8850 | 0.011 |  |  |
| *lpxM* | Val261Gly | missense | 1 | 8850 | 0.011 |  |  |
